# *SoxD* genes are required for adult neural stem cell activation

**DOI:** 10.1016/j.celrep.2022.110313

**Published:** 2022-02-01

**Authors:** Lingling Li, Cristina Medina-Menéndez, Laura García-Corzo, Carmen M. Córdoba-Beldad, Alejandra C. Quiroga, Elena Calleja Barca, Valeriya Zinchuk, Sara Muñoz-López, Pilar Rodríguez-Martín, Maria Ciorraga, Inés Colmena, Silvia Fernández, Carlos Vicario, Silvia K. Nicolis, Véronique Lefebvre, Helena Mira, Aixa V. Morales

**Affiliations:** 1 Instituto Cajal, CSIC, 28002 Madrid, Spain; 2 Instituto de Biomedicina de Valencia, CSIC, 46010 Valencia, Spain; 3 The Children’s Hospital of Philadelphia, Philadelphia, PA 19104, USA; 4 University of Milano-Bicocca, 20126 Milano, Italy; 5 CIBERNED-Instituto de Salud Carlos III, 28029 Madrid, Spain; 6 These authors contributed equally; 7 Lead contact

## Abstract

The adult neurogenic niche in the hippocampus is maintained through activation of reversibly quiescent neural stem cells (NSCs) with radial glia-like morphology (RGLs). Here, we show that the expression of SoxD transcription factors Sox5 and Sox6 is enriched in activated RGLs. Using inducible deletion of *Sox5* or *Sox6* in the adult mouse brain, we show that both genes are required for RGL activation and the generation of new neurons. Conversely, Sox5 overexpression in cultured NSCs interferes with entry in quiescence. Mechanistically, expression of the proneural protein Ascl1 (a key RGL regulator) is severely downregulated in *SoxD*-deficient RGLs, and *Ascl1* transcription relies on conserved Sox motifs. Additionally, loss of Sox5 hinders the RGL activation driven by neurogenic stimuli such as environmental enrichment. Altogether, our data suggest that *SoxD* genes are key mediators in the transition of adult RGLs from quiescence to an activated mitotic state under physiological situations.

## In brief

Li et al. show that Sox5 and Sox6 transcription factors are required for neural stem cell activation and for the generation of new neurons in the adult hippocampus in mice. In that context, they also show that Sox5 and Sox6 modulate the transcription of the stem cell activator *Ascl1*.

## Graphical abstract

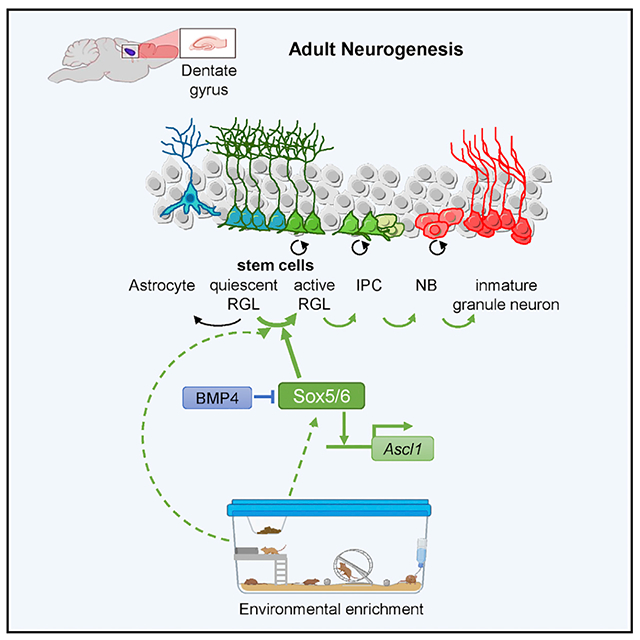

## INTRODUCTION

Neurogenesis, a developmental process generating functionally integrated neurons, occurs throughout life in certain areas of the mammalian brain, such as the ventricular-subventricular zone (V-SVZ) lining the lateral ventricles (Obernier and Alvarez-Buylla, 2019) and the subgranular zone (SGZ) in the dentate gyrus (DG) of the hippocampus (Bond et al., 2015; Goncalves et al., 2016). New neurons produced in the adult SGZ integrate into the adjacent granule cell layer and participate in learning and memory processes (Christian et al., 2014; Goncalves et al., 2016). Additionally, several physiological and pathological situations, such as physical exercise, task learning, environmental enrichment, and seizures, can stimulate neurogenesis in the adult DG (Rolando and Taylor, 2014).

The adult SGZ neurogenic niche is maintained through the activation of neural stem cells (NSCs) with radial glia-like morphology (RGLs). They are mostly in a reversible state of quiescence that protects cells from DNA damage and prevents depletion of the RGL population (Urbán et al., 2019). At any given time, a relatively small population of quiescent RGLs (qRGLs) will activate and will divide symmetrically to self-renew or asymmetrically to generate an RGL and an intermediate progenitor cell (IPC) (Pilz et al., 2018). IPCs generate neuroblasts, which exit the cell cycle to differentiate into granule neurons (GNs) (Christian et al., 2014). However, active RGLs (aRGLs) in the SGZ divide mostly asymmetrically and, on average, will divide a few times before being depleted (Encinas et al., 2011; Pilz et al., 2018) or return to a temporal shallow quiescence or resting state, while most RGLs will remain in a dormant state of deep quiescence (Bottes et al., 2021; Harris et al., 2021). For that reason, a crucial aspect to understand the long-life maintenance of adult neurogenesis is to clarify the mechanisms that control the balance between quiescence and activation in the RGL population.

The transition of RGLs between quiescent and activated states is regulated by local niche signals that promote either quiescence (BMPs, Delta/Notch, and tonic-GABA) (Ehm et al., 2010; Mira et al., 2010; Song et al., 2012) or cell proliferation and neurogenesis (WNT, SHH, and IGF pathways, among others) (Bracko et al., 2012; Favaro et al., 2009; Lie et al., 2005; Nieto-Estevez et al., 2016). Moreover, few of the intrinsic factors linked to the quiescent (Hes5, p57, FOXO3, and REST) or active (TLX and Ascl1) state of NSCs in the adult DG have been identified (Morales and Mira, 2019; Shin et al., 2015). However, little is known about how RGLs integrate signals and intrinsic factors in order to transit from the quiescent to the active/proliferative state.

Sox transcription factors are important players in the control of crucial aspects of adult neurogenesis, such as RGL maintenance (Sox2) (Favaro et al., 2009), IPC proliferation (Sox21) (Matsuda et al., 2012), and newborn neuron maturation (Sox4/Sox11) (Mu et al., 2012). However, the role of members of SoxD subgroup (Sox5, Sox6, and Sox13) in the adult neurogenic niches remains unexplored. Sox5 and Sox6 were previously shown to induce cell cycle exit both in neural precursors during development (Martinez-Morales et al., 2010) and in models of glioblastoma in the adult brain (Kurtsdotter et al., 2017). They are also key in neuronal subtype specification in the developing forebrain and spinal cord (Batista-Brito et al., 2009; Lai et al., 2008; Quiroga et al., 2015). Moreover, *SOX5* and *SOX6* heterozygous inactivating variants cause poorly studied neurodevelopmental diseases in humans (Lamb-Shaffer and Tolchin-Le Caignec syndromes, respectively; OMIM: 616803 and OMIM: 618971).

We show here that SoxD transcription factors are expressed predominantly in RGLs in the adult SGZ and that their expression is inhibited by the quiescence-promoting factor BMP4. Using mice with inducible conditional inactivation of *Sox5* or *Sox6* (Dumitriu et al., 2006; Dy et al., 2008), we show that both SoxD factors are required for *Ascl1* expression in RGLs and are consequently required for RGL activation and new neuron generation in the SGZ.

## RESULTS

### Sox5 and Sox6 are expressed predominantly in RGLs in the adult dentate gyrus

To explore if Sox5 and Sox6 transcription factors (the only two SoxD family members clearly expressed in the adult DG) (Hochgerner et al., 2018) control cell proliferation during adult SGZ neurogenesis, we first characterized their expression pattern in the adult DG. For the sake of clarity, hippocampal stem cells analyzed *in vivo* are called, hereafter, RGLs, while in culture they are referred to as neural stem cells.

**Figure 1.**
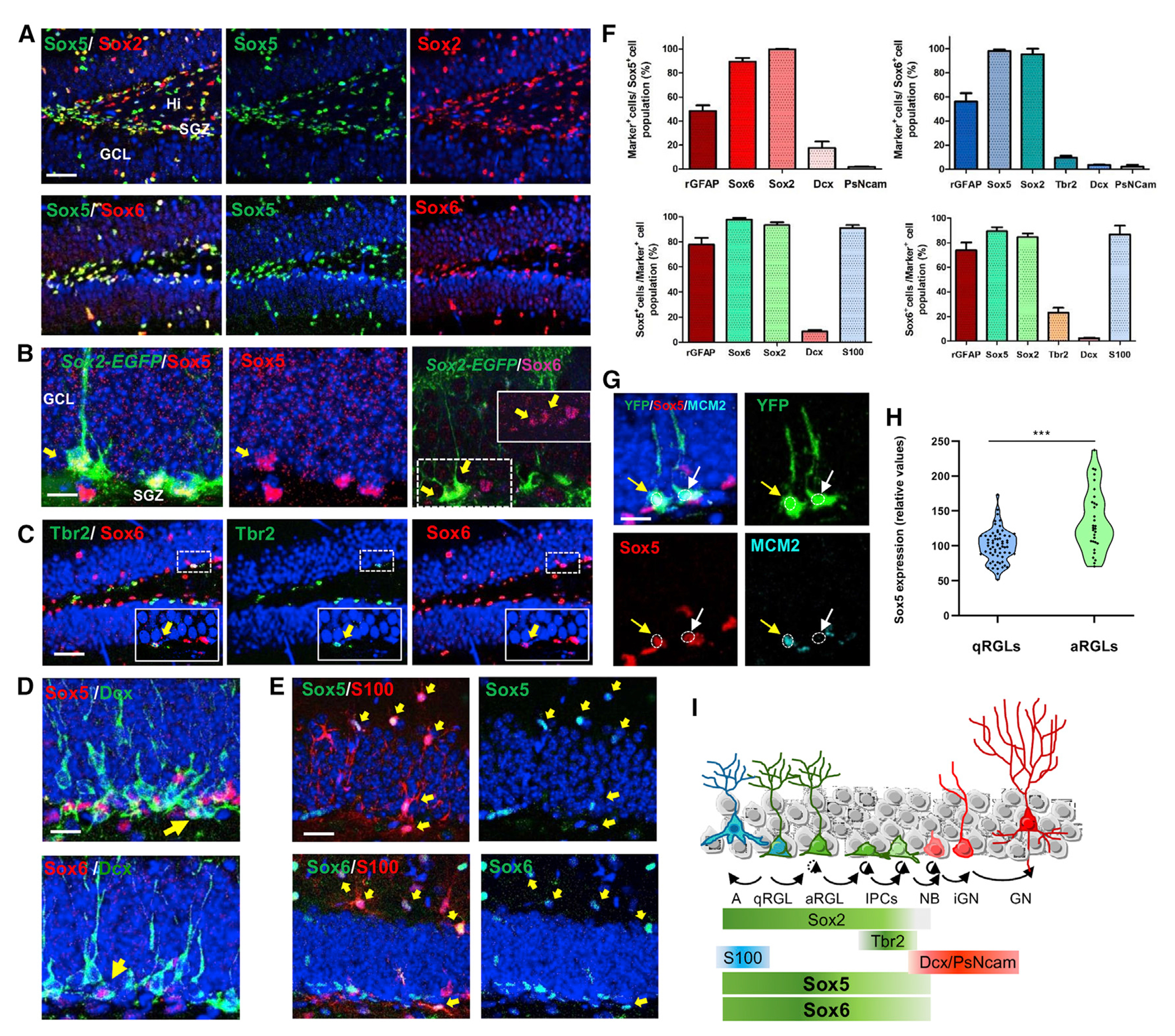
Sox5 and Sox6 are expressed predominantly in RGLs in the adult DG (A and B) Confocal images showing immunohistochemistry with the indicated marker in coronal sections of dorsal DG from 2-month-old mice. (A) Sox5 and Sox6 are co-expressed in the majority of Sox2^+^ cells. (B) Sox5 and Sox6 are expressed in RGLs in *Sox2-EGFP* mice (yellow arrows; higher magnification in box). (C) Sox6 is expressed in a subset of Tbr2^+^ IPCs (higher magnification in boxes). (D) Sox5 and Sox6 are expressed in few Dcx^+^ iGNs (yellow arrows). (E) Sox5 and Sox6 are expressed in S100^+^ astrocytes (yellow arrows). (F) Quantification of the percentage of cells that express the indicated cell type-specific maker in Sox5^+^ cells or Sox6^+^ cells (top; >300 cells/marker) and the percentage of Sox5^+^ or Sox6^+^ cells among cells expressing the indicated cell-type marker (bottom; >300 cells/marker). (G) Confocal images showing RGLs in *Sox2-creER^T2^/Rosa-YFP* mice expressing YFP, MCM2, and Sox5 (yellow arrows). (H) Violin plot representing the intensity of Sox5 immunofluorescence in GFAP^+^MCM2^−^ qRGLs (n = 65) and in GFAP^+^MCM2^+^ aRGLs (n = 31), relative to Sox5 expression levels in qRGLs in *Sox2-creER^T2^/Rosa-YFP* mice. (I) Summary of Sox5- and Sox6-expressing cells in the SGZ. In all panels, nuclei were counterstained with bisbenzimide (blue). n = 3–5 mice and n = 3–6 sections/animal for each immunostaining. A, astrocyte; GN, granular neuron; GCL, granule cell layer; Hi, hilus; iGN, immature granular neuron; IPC, intermediate progenitor cell; SGZ, subgranular zone. Data are represented as mean ± SEM. Unpaired two-tailed Student’s t test: ***p = 1.001 × 10^6^. Scale bars represent 35 μm (A and C), 20 μm (B, D, and E), and 15 μm (G). See also Figure S1.

In 2- to 3-month-old mouse DG, most Sox5- and Sox6-positive cells co-expressed Sox2 (99.6% ± 0.2% and 96.3% ± 2.4%, respectively; Figures 1A and 1F). Similarly, the vast majority of Sox2^+^ cells expressed Sox5 and Sox6 (93.2% ± 2.5% and 84.6% ± 3.1%; Figures 1A and 1F). Moreover, 89.3% ± 3.2% of Sox5^+^ cells expressed Sox6, and 97.9% ± 1.3% of Sox6^+^ cells expressed Sox5 (Figures 1A and 1F). These data indicate that Sox5, Sox6, and Sox2 proteins are mostly co-expressed in cells of the SGZ in the adult hippocampus.

As the Sox2^+^ cell population includes both RGLs and IPCs with non-radial morphology (Suh et al., 2007), we used a transgenic *Sox2-EGFP* reporter mouse line, in which RGLs could be recognized by the radial processes of GFP-filled cells extending toward the molecular layer. We observed that the vast majority of GFP^+^ RGLs expressed both Sox5 and Sox6 (Figure 1B). Using GFAP staining, we confirmed that around half of Sox5^+^ and Sox6^+^ cells showed radial morphology and GFAP expression (rGFAP; 48.5% ± 4.6% and 56.2% ± 6.9%, respectively; Figure 1F) and that the majority of rGFAP^+^ cells express Sox5 or Sox6 (77.8% ± 5.5% and 73.8% ± 6.1%, respectively; Figure 1F).

To further analyze Sox5 expression in the RGL population, we used *Sox2-creER*^*T2*^/*Rosa26-YFP* mice injected for 5 days with tamoxifen (TAM) to induce creER^T2^ nuclear translocation leading to YFP activation via cre-mediated recombination, as the forebrain enhancer of *Sox2* drives cre-recombinase expression mostly in RGLs (Favaro et al., 2009) (Figure 1G). We observed significantly lower Sox5 expression in non-proliferating quiescent qRGLs (rGFP^+^MCM2^−^ cells; MCM2 is a marker for proliferating cells) in comparison with that found in proliferating active aRGLs (rGFP^+^MCM2^+^ cells; relative levels of 100.0 ± 2.83 versus 135.00 ± 7.90, respectively; Figures 1G and 1H). However, we did not observe any significant change in Sox6 levels when comparing qRGLs and aRGLs (Figure S1). These data indicate that *SoxD* genes are expressed in adult hippocampal RGLs, and at least Sox5 is enriched in activated RGLs.

In the adult DG, only a small fraction of aRGLs undergo neurogenic division to generate IPCs, which ultimately differentiate into GNs (Christian et al., 2014). We found that only a fraction of Tbr2^+^ IPCs expressed Sox6 (23.0% ± 3.9%; Figures 1C and 1F) and probably Sox5. Finally, only a minority of postmitotic immature GNs (Dcx^+^ or PSA-NCAM^+^) expressed Sox5 (8.6% ± 1.3%; Figures 1D and 1F) or Sox6 (2.6% ± 0.3%; Figures 1D and 1F). Finally, using S100 immunostaining to identify astrocytes, we found that almost all DG astrocytes expressed Sox5 (91.1% ± 2.3%; Figures 1E and 1F) and Sox6 (86.7% ± 7.2%; Figures 1E and 1F).

In summary, Sox5 and Sox6 are co-expressed in hippocampal RGLs, with Sox5 expression being higher in aRGLs. Their expression is maintained in IPCs, but progressively lost as IPCs exit the cell cycle and differentiate into GNs (summarized in Figure 1I).

### Sox5 and Sox6 are required for the activation of qRGLs in the adult DG

**Figure 2.**
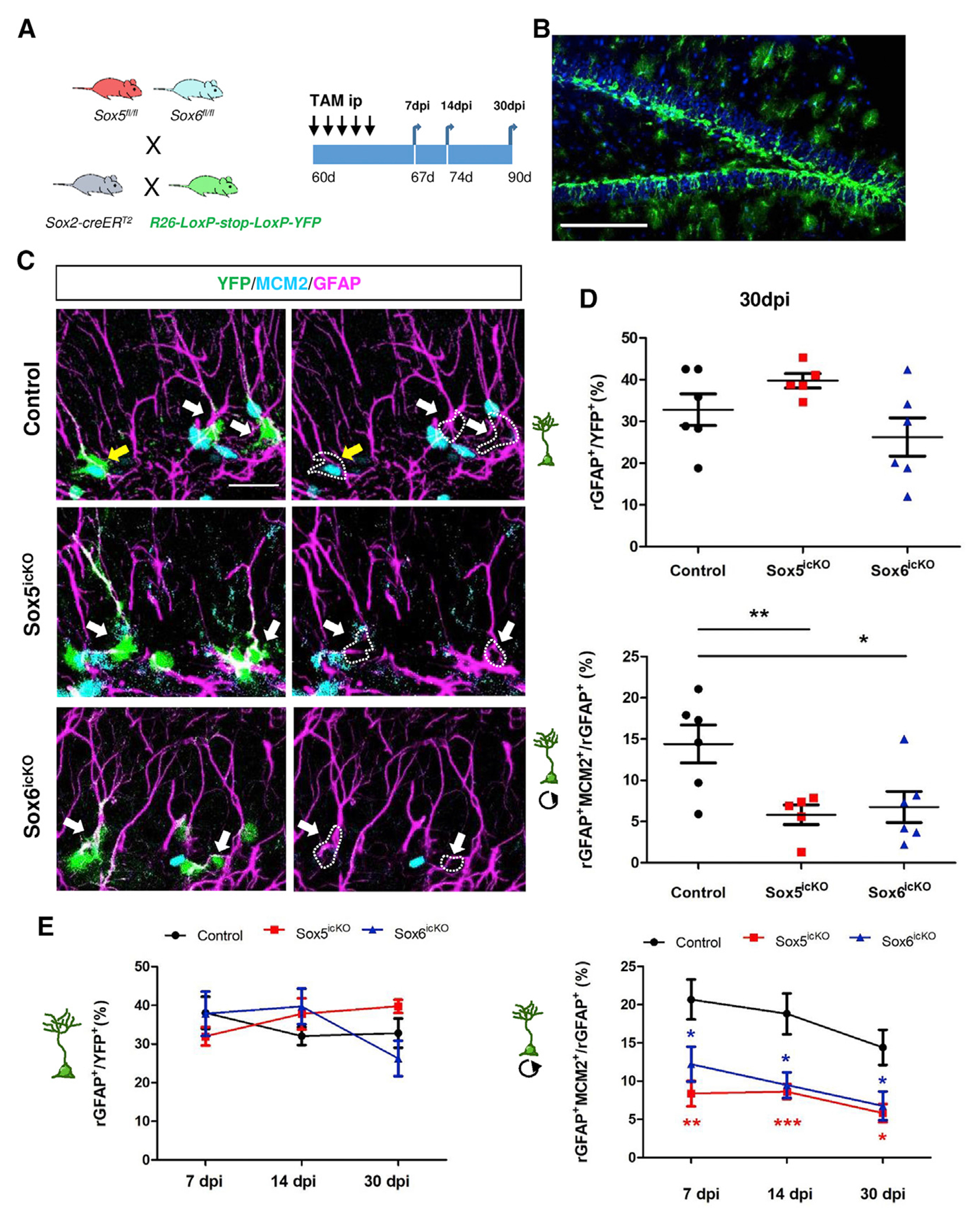
Sox5 and Sox6 are required for the activation of adult RGLs (A) Generation of mice harboring a *Sox5* or *Sox6* inducible conditional null allele in the adult brain. TAM intraperitoneal (i.p.) injection was performed in 2- to 3-month-old mice on 5 consecutive days, and after 7, 14, or 30 days post-TAM injection (dpi), brains were collected. (B) Confocal image showing YFP^+^ cells that underwent recombination 30 dpi in a control mouse (Sox2-creER^T2^/Rosa-YFP). (C) Confocal images showing YFP, MCM2, and radial GFAP (rGFP) at 30 dpi in adult control, Sox5^icKO^, and Sox6^icKO^ mice. White arrows indicate YFP^+^rGFAP^+^MCM2^−^ cells and yellow arrows YFP^+^rGFAP^+^MCM2^+^ cells. (D) Quantitation of the number of rGFAP^+^ cells among YFP^+^ cells and of proliferating rGFAP^+^MCM2^+^ cells among rGFAP^+^YFP^+^ cells in the indicated mice at 30 dpi. Each symbol represents an independent biological replicate. (E) Quantitation of the number of rGFAP^+^ cells in YFP^+^ cells and proliferating rGFAP^+^MCM2^+^ cells in rGFAP^+^ YFP^+^ cells in mice at 7dpi (n = 5, 4, and 4 for control, Sox5^icKO^, and Sox6^icKO^, respectively), 14 dpi (n = 5, 9, and 6) and 30 dpi (n = 6, 5, and 6). Data represent mean value ± SEM. n > 100 cells for each quantification. Unpaired two-tailed Student’s t test comparing each group with control: *p < 0.05, **p < 0.01, and *** p < 0.001. Scale bars, 100 μm (B) and 20 μm (C). See also Figure S3.

Having shown that *SoxD* genes are expressed in RGLs, we next asked whether they have important functions in neurogenesis in the adult hippocampus. For that purpose, we adopted a genetic loss-of-function approach coupled to fate mapping. We generated mice harboring *Sox5* and/or *Sox6* conditional null alleles (*Sox5*^*fl/fl*^ and/or *Sox6*^*fl/fl*^) (Dumitriu et al., 2006; Dy et al., 2008), a transgene expressing a tamoxifen-inducible form of cre-recombinase (CreER^T2^) under the control of a forebrain *Sox2* enhancer (*Sox2-cre-ER*^*T2*^) (Favaro et al., 2009), and a *Rosa26-floxed stop-YFP* reporter allele (R26-YFP; Figure 2A) (Srinivas et al., 2001). These mice were named Sox5^icKO^ and/or Sox6^icKO^. R26-YFP allowed fate mapping of RGL cells that had undergone selective *Sox5* or *Sox6* deletion (Figure 2B), and all analyses were done exclusively in YFP^+^ cells. *Sox2-CreER*^*T2*^ mice carrying the R26-YFP reporter transgene, but wild-type or heterozygous for *Sox5*^*fl*^ or *Sox6*^*fl*^, were used as controls. Two-month-old control and mutant mice were injected for 5 consecutive days with TAM and analyzed 7, 14, and 30 days after the first TAM injection (dpi; Figure 2A).

First, we evaluated the efficiency of the TAM-induced knockout at 7 dpi. Compared with control mice, there was a marked decrease in the number of *Sox5*^+^*YFP*^+^ cells in Sox5^icKO^ mice (53.8% ± 3.9% versus 89.3% ± 2.2% in control, considering 100% as the total number of YFP^+^ recombined cells; p = 0.0002; Figures S2A and S2D). Similarly, the number of Sox6^+^YFP^+^ cells in Sox6^icKO^ mice was clearly reduced after TAM injection (33.7% ± 3.0% versus 75.9% ± 1.1% in control; p = 0.000006; Figures S2B and S2D). Although a fraction of YFP^+^ cells had failed to recombine all *Sox5*^*fl*^ and *Sox6*^*fl*^ alleles, we observed significant alterations in the neurogenic niche, as we describe below. We discarded the possibility of analyzing double Sox5^icKO^/Sox6^icKO^ mice, as we estimated that only 3% of recombined YFP^+^ cells had lost both Sox5 and Sox6 expression (Figure S2E).

As Sox2 is a transcription factor essential for RGL stemness, we checked its expression and did not find any differences in YFP^+^ cells in control, Sox5^icKO^, or Sox6^icKO^ mice (Figures S2A–S2C). These results indicate that Sox2 expression does not depend on Sox5 or Sox6 in the adult SGZ. Moreover, at 7 dpi the proportions of RGLs among YFP^+^ cells in Sox5^icKO^ or Sox6^icKO^ mice were similar to those in control mice (32.0% ± 2.4% and 37.8% ± 5.7%, respectively, versus 38.1% ± 4.1%; Figures 2E, S3A, and S3B), indicating that despite the loss of Sox5 or Sox6, the recombined RGL pool is maintained for at least 1 week.

In contrast, the fraction of proliferating MCM2^+^rGFAP^+^ cells was significantly reduced in Sox5^icKO^ and Sox6^icKO^ mice compared with that in control mice (8.4% ± 1.7% and 11.9% ± 2.1, respectively, versus 20.7% ± 2.6% in control; p = 0.0075 and p = 0.0400; Figures 2E, S3A, and S3B). These data demonstrate that loss of Sox5 or Sox6 prevented adult RGL activation after 7 days.

We further investigated the activation of RGLs in mutant animals at 14 and 30 dpi. Compared with control mice, the proportion of rGFAP^+^ cells among the YFP^+^ population did not change in Sox5^icKO^ or Sox6^icKO^ mice 14 dpi (37.8% ± 4.0% and 39.7% ± 4.6%, respectively, versus 32.1% ± 2.3% in control; Figures 2E and S3C) or 30 dpi (39.8% ± 1.7% and 26.3% ± 4.6%, respectively, versus 32.8% ± 3.8% in control; Figures 2C–2E). However, the proportion of proliferating MCM2^+^ cells in the RGL population at 14 dpi decreased to ~50% in both Sox5^icKO^ and Sox6^icKO^ mice (8.9% ± 1.1% and 9.5% ± 1.7%, respectively versus 18.8% ± 2.7%; p = 0.0009 and p = 0.0181; Figures 2E and S3C). This reduction was clearly maintained at 30 dpi in Sox5^icKO^ and Sox6^icKO^ mice (5.8% ± 1.2% and 6.8% ± 1.9%, respectively versus 14.4% ± 2.3% in control; p = 0.0100 and p = 0.0237; Figures 2C–2E). Taken together, these data indicate that Sox5 and Sox6 are required for the activation of RGLs in the adult DG from 7 to 30 days after cre-mediated activation of gene deletion.

**Figure 3.**
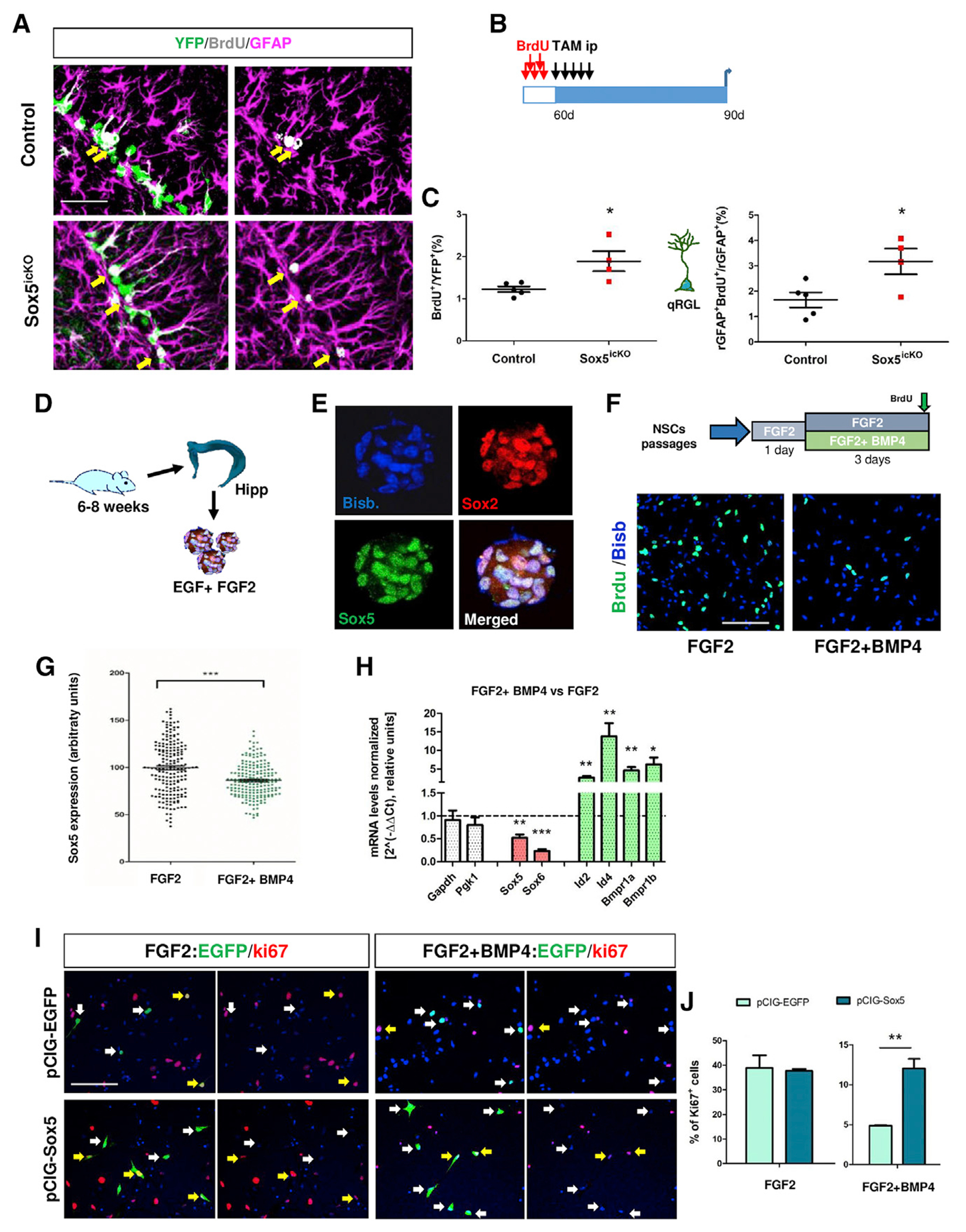
Loss of Sox5 favors quiescent state in RGLs and BMP4 inhibit Sox5 expression (A) Confocal images showing immunostaining for YFP, BrdU, and GFAP of control and Sox5^icKO^ mice brain sections at P90 at 30 dpi. Yellow arrows indicate recombined RGL cells that retain BrdU after 1 month (YFP^+^ BrdU^+^ rGFAP^+^ cells). (B) Experimental design for BrdU label retention experiment. (C) Quantification of BrdU^+^ cells among YFP^+^ recombined cells and BrdU^+^ rGFAP^+^ YFP^+^ cells among rGFAP^+^ YFP^+^ cells. Each symbol represents an independent biological replicate. (D) Scheme of adult NSC preparation grown as neurospheres. (E) Immunohistochemistry in hippocampal neurospheres for Sox2 and Sox5. (F) Neurospheres dissociated and seeded as attached cells and cultured with FGF2 alone or in combination with BMP4. A 40 min BrdU pulse was given before fixation, and BrdU incorporation was analyzed using immunohistochemistry. (G) Quantitation of Sox5 protein levels in NSCs in FGF2 and in FGF2 plus BMP4 conditions expressed in arbitrary units relative to the FGF2 condition. p = 0.0001 by Student’s t test (n = 300 cells). (H) qPCR analysis for the indicated transcripts in NSCs grown with FGF2 plus BMP4 with respect to those grown with FGF2 alone. Results are shown as 2^−ΔΔCt^ normalized with respect to two housekeeping genes (*Pgk1* and *Gapdh*) and relative to values in cells grown in FGF2 alone (dashed line on y axis = 1). A total of three or four independent neurospheres cultures were used, and three technical replicates were performed. (I) Immunohistochemistry of nucleofected NSCs (YFP^+^ cells; green), using either pCIG or pCIG-Sox5 vectors, to analyze proliferating Ki67^+^ cells in each condition. (J) Quantitation of the percentage of Ki67^+^ cells in each indicated condition. In all graphs, data are mean value ± SEM. *p < 0.05, **p < 0.01, and ***p < 0.001 by unpaired (C) or paired (H and J) Student’s t test. Scale bars represent 50 μm (A), 30 μm (E), and 80 μm (F and I).

We next explored whether the reduction in adult RGL activation upon Sox5 deletion was accompanied by a complementary increase in qRGLs. Dividing RGLs that return to quiescence can be identified by their capacity to incorporate BrdU during DNA replication and then to retain this thymidine analog for as long as they are quiescent (BrdU-long [BrdU-L] retaining RGLs). They can be labeled in P60 mice by five consecutive BrdU injections and, upon *Sox5* inactivation, be chased for 30 days (Urbán et al., 2016; Figure 3B). At P90, the percentage of BrdU-L-retaining cells was larger in Sox5^icKO^ than in control mice (1.9% ± 0.2% versus 1.2% ± 0.07%; p = 0.020; Figures 3A and 3C). Moreover, Sox5^icKO^ mice showed twice as many quiescent BrdU-L/rGFAP^+^ cells as control mice (3.2% ± 0.5% versus 1.7% ± 0.3%; p = 0.029; Figures 3A and 3C). This pool of BrdU-L RGLs probably corresponds to resting RGLs with shallow quiescence (those more prone to resume cell cycle entry), in contrast with dormant RGLs in a deeper state of quiescent (Harris et al., 2021). Our data indicate that in the absence of Sox5, those resting RGLs show impaired ability to re-enter the cell cycle and remain quiescent. In summary, these data reinforce the fact that Sox5 is required for RGL transition from quiescence to activation in the adult hippocampal SGZ.

### Sox5 levels are regulated by BMP4 and Sox5 overexpression is sufficient to interfere with entry in quiescence *in vitro*

To further prove that Sox5 is required in adult RGLs for the transition from quiescence to activation, we used an *in vitro* model, in which hippocampus-derived NSCs from 6-week-old mice are grown as floating Sox5- and Sox2-positive neurospheres in the presence of the EGF and FGF2 mitogens (Figure 3D).

One of the best established signals that promote entry in the quiescent state is BMP4. This factor is active both in hippocampal RGLs *in vivo* (Bonaguidi et al., 2008; Mira et al., 2010) and in hippocampal NSCs *in vitro* (Blomfield et al., 2019; Martynoga et al., 2013; Mira et al., 2010). Hippocampal neurospheres were disaggregated and seeded as adherent cells and cultured with FGF2 alone (proliferating condition) or combined with BMP4 (quiescence condition; Figure 3F). As expected, 3 times as few NSCs incorporated BrdU when cultured with FGF2 plus BMP4 as with FGF2 alone (21.2% ± 3.2% and 6.9% ± 0.4%; p = 0.0016; Figure 3F). Moreover, to validate our approach by real-time quantitative PCR (qPCR), we observed an increase in transcriptional activation of BMP4 targets such as *Id2* (2.6 ± 0.5-fold, p = 0.011), *Id4* (13.8 ± 3.5-fold, p = 0.0073) (Blomfield et al., 2019), *Bmpr1a* (4.6 ± 0.9-fold, p = 0.0087), and *Bmpr1b* (6.2 ± 1.8-fold, p = 0.0406) in comparison with NSCs grown in FGF2 alone (Figure 3H). Interestingly, in BMP4-induced quiescent NSCs, *Sox5* and *Sox6* transcript levels were significantly reduced (0.52 ± 0.07-fold and 0.23 ± 0.04-fold, respectively; p = 0.0027 and p = 0.00004; Figure 3H) in comparison with values in proliferating conditions. Similarly, at the protein level, Sox5 expression was significantly decreased in cells in BMP4-induced quiescence in comparison with actively proliferating cells (relative level 86.19 ± 1.28 versus 99.57 ± 1.20, p = 0.001; Figure 3G). These data suggest that the BMP4 signal, which strongly promotes the quiescent state in NSCs, inhibits *Sox5* and *Sox6* expression at the mRNA level and consequently at the protein level as well.

Having seen that loss of Sox5 blocks the activation of RGLs *in vivo*, we decided to check if Sox5 was sufficient to promote NSC activation *in vitro*. For that purpose, we used hippocampal NSCs and overexpressed Sox5 (via a pCIG-Sox5 construct) through nucleofection, using pCIG-EGFP nucleofected cells as control. In the FGF2 condition, we observed similar number of proliferative Ki67^+^ cells (among EGFP-expressing cells) in both control and Sox5-overexpressing cells (38.90% ± 5.13% versus 37.70% ± 0.71%, respectively; Figures 3I and 3J), suggesting that increased levels of Sox5 did not alter NSC proliferation. However, in the FGF2 plus BMP4 condition, cells overexpressing Sox5 were more proliferative than control cells (12.03% ± 1.21% versus 4.86% ± 0.09%, respectively; p = 0.0041; Figures 3I and 3J). Thus, our data suggest that Sox5 overexpression is sufficient to interfere with the entry of NSCs into BMP4-induced quiescence.

### Sox5 and Sox6 are required for neurogenesis in the adult DG

**Figure 4.**
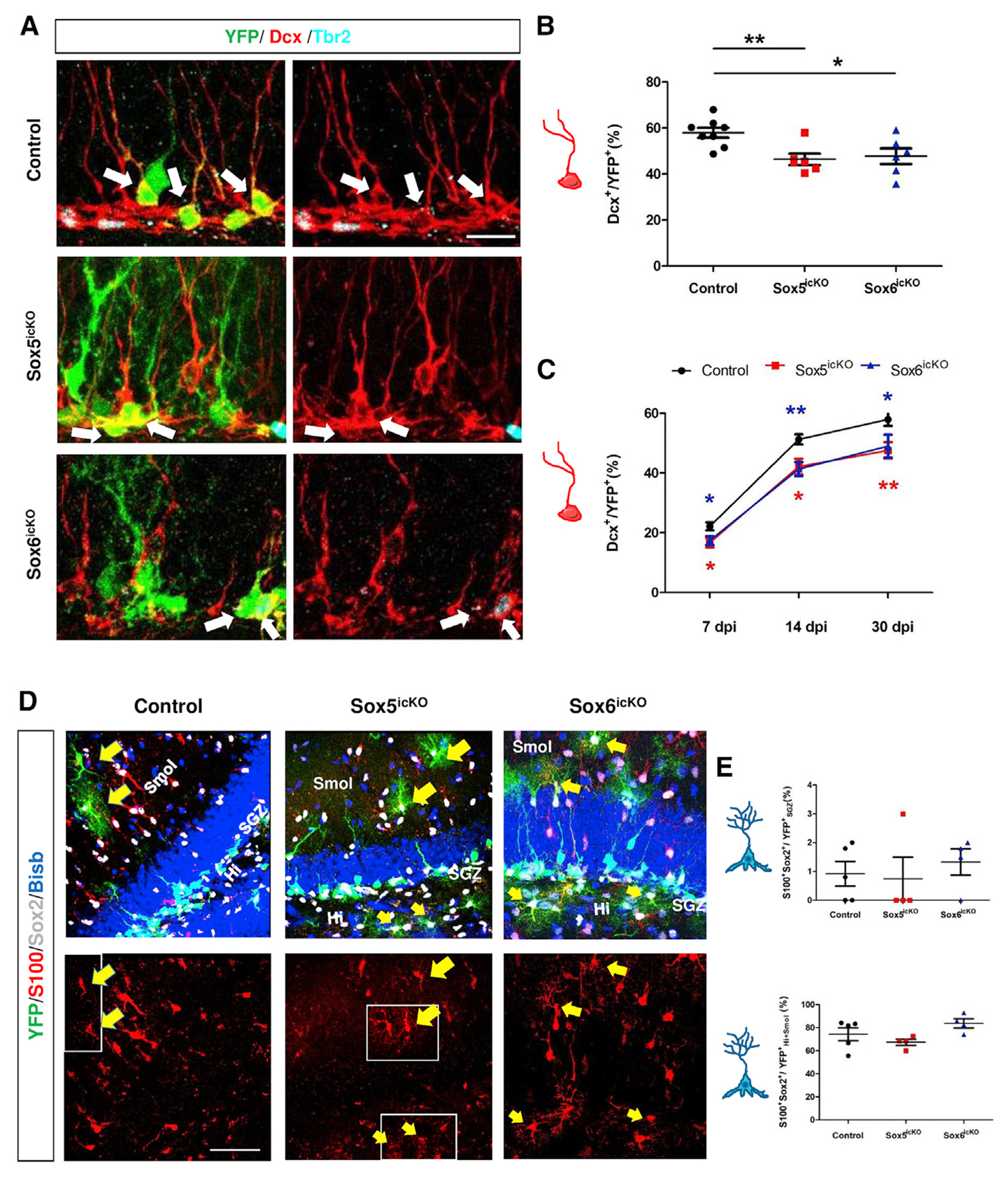
SOX5 and Sox6 are required for the generation of newborn neurons and are not essential for astrogliogenesis (A) Confocal images showing YFP, Dcx, and Tbr2 in the SGZ at 30 dpi in 3-month-old control, Sox5^icKO^, and Sox6^icKO^ mice. White arrows indicate Dcx^+^ YFP^+^ cells. (B) Quantitation of Dcx^+^ cells number among YFP^+^ cells in the indicated mice. (C) Comparison of the number of Dcx^+^ cells among YFP^+^ cells in control, Sox5^icKO^, and Sox6^icKO^ mice at 7 dpi (n = 8, 6, and 7 for control, Sox5^icKO^, and Sox6^icKO^, respectively), 14 dpi (n = 7, 7, and 6), and 30 dpi (n = 8, 6, and 6). (D) Analysis of astrogliogenesis in YFP^+^ cells of 3-month-old control, Sox5^icKO^, and Sox6^icKO^ mice at 30 dpi using immunohistochemistry for S100 and Sox2 to identify astrocytes. Yellow arrows indicate YFP^+^S100^+^Sox2^+^ astrocytes. White boxes correspond to single z planes to better visualize S100 expression in some cells. (E) Quantitation of the number of S100^+^Sox2^+^ astrocytes in the recombined YFP+ cells in the SGZ (top) or the hilus (Hi) and stratum moleculare (Smol) zones (bottom) in the indicated mice. SGZ, subgranular zone. Data are mean value ± SEM. *p < 0.05, **p < 0.01, and ***p < 0.001 by unpaired Student’s t test. Scale bars represents 15 μm (A), 50 μm (D, left and center), and 40 μm (D, right). See also Figure S4.

To determine if the reduction in RGL proliferation in Sox5^icKO^ and Sox6^icKO^ mice had any consequence in the generation of new neurons in the adult DG, IPCs and immature neurons were analyzed. At 7 dpi after inducing *Sox5* and/or *Sox6* deletion, a reduction of ~35% in the number of IPCs expressing Tbr2 and YFP was detected in Sox5^icKO^ and Sox6^icKO^ mice (16.4% ± 2.1% [p = 0.007] and 17.0% ± 1.6% [p = 0.004], respectively; Figures S4A and S4B) relative to control mice (25.8% ± 1.8%). Despite this short-term analysis, mutants showed a ~25% reduction in Dcx^+^YFP^+^ cells relative to control mice (16.7% ± 1.6% [p = 0.02] and 17.4% ± 1.5% [p = 0.03], respectively, with respect to control [22.2% ± 1.3%]; Figures 4C, S4A, and S4B). Thus, both Sox5 and Sox6 are required for short-term generation of new neurons in the hippocampal dentate gyrus.

We further investigated adult neurogenesis in animals at 14 and 30 dpi to determine the mid-term effect of Sox5 and Sox6 loss in the generation of new neurons. Although the number of Tbr2^+^ cells did not change much in Sox5^icKO^ and Sox6^icKO^ mice at 14 and 30 dpi (Figure S4C), the proportion of Dcx^+^YFP^+^ new neurons decreased by ~18% in relation to control mice 14 dpi (42.6% ± 3.1% and 41.4% ± 2.3%, respectively, versus 51.3 ± 1.7% in control; p = 0.015 and p = 0.005; Figures 4C and S4C). The reduction in the generation of new neurons was maintained at 30 dpi, when we observed a ~20% decrease in the fraction of immature Dcx^+^YFP^+^ neurons in Sox5^icKO^ and Sox6^icKO^ mice compared with control mice (46.4% ± 2.5% [p = 0.004] and 47.8% ± 3.4% [p = 0.02], respectively, versus 58.0% ± 2.2% in control; Figures 4A–4C). Taken together, our data demonstrate that in the adult SGZ, Sox5 and Sox6 are both required for the generation of the correct number of new granular neurons.

However, in addition to neurogenesis, new astrocytes are continuously generated from RGLs in the adult DG (Bonaguidi et al., 2011; Encinas et al., 2011), and in some mutant mice, the astrogliogenic fate is promoted at the expenses of a neurogenic one (Bonzano et al., 2018). We found that as early as 7 dpi, Sox2-creER^T2^-mediated recombination in control mice could be observed in S100^+^Sox5^+^ astrocytes in the hilus (Hi) and stratum moleculare (Smol; Figure S5A) of the hippocampus. By 30 dpi, in both Sox5^icKO^ and Sox6^icKO^ mice, the number of S100^+^ Sox2^+^ astrocytes in the YFP^+^ population in the SGZ was similar (0.75% ± 0.75% and 1.33% ± 0.45%; Figures 4D and 4E) to that in control mice (0.92% ± 0.43%). The number of astrocytes in the YFP^+^ population recombined in Hi and Smol was also similar in Sox5^icKO^ and Sox6^icKO^ mice (67.4% ± 2.7% and 83.8% ± 3.9%, respectively; Figures 4D and 4E) with respect to control mice (74.3% ± 5.7%). Thus, in control, Sox5^icKO^, and Sox6^icKO^ mice, the fraction of Sox2-creER^T2^-mediated recombined YFP^+^ cells that become astrocytes in the SGZ is very low (~1%), and Sox5 or Sox6 are probably not required for the generation of astrocytes in the DG area, at least in young animals.

Finally, to discard further the option that Sox5 and Sox6 promote astrogliogenesis, we used hippocampal NSCs in culture from Sox5^icKO^ and Sox6^icKO^ mice, administered 4-OH-TAM to induce creER^T2^-mediated recombination, and then forced NSCs to differentiate upon mitogen withdrawal (Figure S5B). We used βIII-tubulin and GFAP immunostaining to characterize neurons and astrocytes, respectively. Loss of Sox5 induced higher neuronal differentiation in YFP^+^ cells with respect to YFP^−^ cells (31.1% ± 5.9% versus 12.4% ± 2.7%, p = 0.029; n = 4; Figures S5C and S5D). In contrast, there was a slight, although not statistically significant, decrease in astrocytic differentiation (8.2% ± 1.9% in YFP^+^ cells versus 12.7% ± 1.2% in YFP^−^ cells, p = 0.09; Figures S5C and S5D). Similarly, loss of Sox6 promoted neuronal differentiation more frequently in YFP^+^ than in YFP^−^ cells (29.3% ± 4.7% versus 15.6% ± 1.6%, respectively; p = 0.03; n = 4; Figures S5C and S5E) but did not significantly affect astrocytic differentiation (3.7% ± 1.4% in YFP^+^ cells versus 11.6% ± 3.5% in YFP^−^ cells, p = 0.08; Figures S5C and S5E). Thus, these data suggest that in NSCs grown in differentiating conditions, Sox5 and Sox6 are probably not essential for astrocytic differentiation and that loss of Sox5 or Sox6 could promote neuronal cell fate. This fact also reinforces the idea that *in vivo*, the main cause of the reduction in neurogenesis in Sox5^icKO^ and Sox6^icKO^ mice is the loss of activation of RGLs, not an alteration in neuronal cell fate commitment or in neuronal differentiation.

### Sox5 and Sox6 control *Ascl1* expression in RGLs

**Figure 5.**
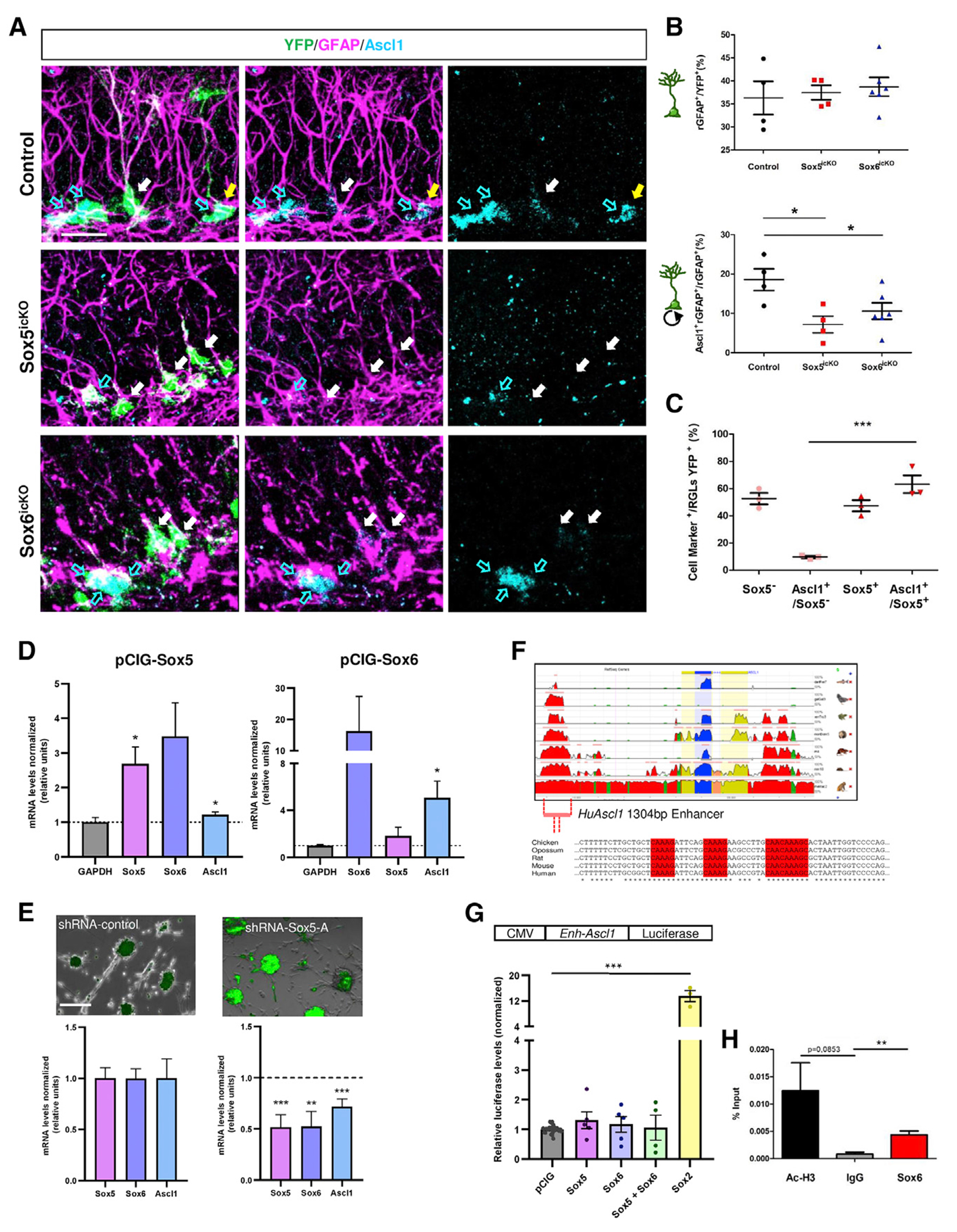
SoxD factors control *Ascl1* expression in RGLs and NSCs of the hippocampal neurogenic niche (A) Confocal image showing immunostaining for YFP, Ascl1, and GFAP in the SGZ of 3-month-old control, Sox5^icKO^, and Sox6^icKO^ mice at 14 dpi. Cyan arrows indicate recombined Ascl1^+^ rGFAP^−^ cells, white arrows rGFAP^+^ cells, and yellow arrows double rGFAP^+^ Ascl1^+^ cells. (B) Quantitation of the number of rGFAP^+^ in the population of YFP^+^ cells and of rGFAP^+^ Ascl1^+^ cells in the rGFAP^+^ YFP^+^ group in the indicated mice. (C) Quantitation in Sox5^icKO^ mice of the proportion of YFP^+^ RGLs maintaining or losing Sox5 expression (Sox5^+^ or Sox5^−^, respectively). The percentage of Ascl1^+^ cells in the subset of Sox5^+^/YFP^+^ or Sox5^−^/YFP^+^ RGLs is indicated. (D) Transcription levels for the indicated genes analyzed using qPCR in hippocampal NSCs transfected with Sox5-pCIG or Sox6-pCIG, represented as 2^−ΔΔCt^ normalized with respect to the housekeeping gene *Gapdh* and relative to values in NSCs transfected with control pCIG (dashed line on y axis = 1). (E) Representative images of NSCs transduced with lentiviral particles expressing shRNA-control or shRNA-Sox5-A and GFP as a reporter protein. Graphs showing transcriptional levels by qPCR for each lentiviral transduced condition. Data are represented as 2^−ΔΔCt^ normalized with respect to the housekeeping gene *Gapdh* and relative to values in NSCs transfected with shRNA-control. (F) Genomic sequence alignment comparing human *ASCL1* locus with homologous *D. rerio, G. gallus, X. tropicalis, M. domestica, R. norvegicus, M. musculus*, and *M. rhesus Ascl1* loci. Presence of conserved Sox-binding sites (red squares) within a 1,304 bp conserved enhancer, 6.6 kb upstream of the human *ASCL1* coding region. (G) Quantitative analysis of transcriptional activity of Sox5, Sox6, and Sox2 on a 1 kb mouse *Ascl1* enhancer in adult NSCs. Graph shows normalized luciferase activity units relative to the pCIG control. Dots in bars correspond to independent experiments. (H) Quantitation of three independent experiments of ChIP assays in rat adult hippocampal NSCs. DNA fragments for *Ascl1* immunoprecipitated with Ac-H3, IgG, or anti-SOX6 antibodies were analyzed using qPCR with primers specific for a 214 bp fragment containing Sox-binding sites depicted in (F). Data are expressed as percentage of input = 100 × 2^[adjusted input – Ct (IP)]^. Results are shown as mean value ± SEM. *p < 0.05, **p < 0.01, and ***p < 0.001 by unpaired Student’s t test. Scale bars, 25 μm (A) and 50 μm (D). See also Figure S6.

One of the most relevant factors in the control of adult RGL activation is Ascl1, a bHLH transcription factor required for the exit of RGLs from quiescence (Andersen et al., 2014). To explore a possible molecular mechanism by which Sox5 or Sox6 could control RGL activation, we analyzed Ascl1 expression in the SGZ of Sox5^icKO^ and Sox6^icKO^ mice at 14 dpi. As previously mentioned, at 14 dpi the relative number of YFP^+^ RGLs expressing rGFAP was similar in Sox5^icKO^, Sox6^icKO^, and control mice (Figures S3C, S5A, and S5B). However, we observed a clear reduction in the proportion of Ascl1^+^rGFAP^+^ cells in the rGFAP^+^YFP^+^ population both in Sox5^icKO^ and Sox6^icKO^ mice (7.2% ± 2.1% [p = 0.017] and 10.6% ± 2.1% [p = 0.046], respectively) with respect to those in control mice (18.6% ± 2.8%; Figures 5A and 5B). These data indicate that Sox5 and Sox6 are required for the expression of Ascl1 in RGLs in the adult hippocampal SGZ.

Additionally, because in Sox5^icKO^ mice only 52.6% ± 5.9% of recombined YFP^+^ RGLs had lost Sox5 (Sox5^−^; Figure 5C), whereas the remaining 47.4% ± 5.9% of YFP^+^ RGLs maintained Sox5 expression (Sox5^+^; Figure 5C), we checked in more detail Ascl1 expression in both RGL populations. We found that whereas 63.2% ± 9.2% of Sox5^+^YFP^+^ RGLs express Ascl1, only 9.7% ± 1.0% of Sox5^−^YFP^+^ RGLs retain Ascl1 expression (p = 0.0012; Figures 5C and S6). These results point to a dependency of Ascl1 expression on Sox5 transcription factor.

Moreover, using lentiviral particles to downregulate Sox5 expression in adult hippocampal NSCs, we observed that a specific short hairpin RNA (shRNA)-Sox5 produced a consistent downregulation in Sox5 levels with respect to shRNA-control scrambled (0.5 ± 0.1-fold with shRNA-Sox5-A versus shRNA-control; set = 1.00; p = 0.004; Figure 5E). Reduced levels of Sox5 provoked a moderate reduction in *Ascl1* (0.7 ± 0.1-fold with shRNA-Sox5-A versus shRNA-Control; set = 1.00; p = 0.009) and in *Sox6* transcription (0.5 ± 0.1-fold with shRNA-Sox5-A versus shRNA-control; set = 1.00; p = 0.02). These results support the conclusion that Sox5 is required for *Ascl1* transcription in adult NSCs *in vitro*.

To determine if Sox5 and Sox6 were sufficient to drive *Ascl1* transcription, we transfected adult NSCs with either pCIG-Sox5 (2.7 ± 0.4-fold *Sox5* overexpression) or pcIG-Sox6 (16.2 ± 9.1-fold *Sox6* overexpression; Figure 5D). In both cases, SoxD overexpression provoked an increase in *Ascl1* levels with respect to control pCIG nucleofected cells (1.2 ± 0.1-fold and 5.1 ± 1.2-fold versus control; set = 1.00; p = 0.040 and p = 0.045, respectively; Figure 5D). In those experiments, there were not clear transcriptional cross regulation of other *SoxD* gene, as *Sox6* or *Sox5* expression was not altered upon Sox5 or Sox6 overexpression (3.5 ± 0.8 and 1.8 ± 0.7, respectively; p = 0.064 and p = 0.328; Figure 5D), and *Sox13* was not at detectable levels in adult hippocampal NSCs. Thus, both Sox5 and Sox6 induce *Ascl1* transcription in adult hippocampal NSCs.

Nevertheless, the loss of Ascl1 expression could be either the cause or the consequence of the loss of RGL activation in Sox5^icKO^ and Sox6^icKO^ mice. To explore the possibility that Sox5 and Sox6 could directly control *Ascl1* expression, we examined putative regulatory sequences near the *Ascl1* genomic locus. An evolutionarily conserved region of 1,304 bp, located 6.6 kb upstream of the human *ASCL1* coding region, was detected when fish, frog, chicken, opossum, rodent, and monkey *Ascl1* locus sequences were compared (hg19 chr12:103345375-103346678; ECR Genome Browser; Figure 5F). An equivalent mouse *Ascl1* coding region drives central nervous system reporter gene expression in transgenic mice (Verma-Kurvari et al., 1998).

Using transcriptional binding sites analysis (Regulatory VISTA, Mulan alignment, and bibliography mining), we uncovered three highly conserved putative Sox-binding sites (G/_A_ A/_G_ ACAA T/_A_ G/_A_ G/_C_) (Lee et al., 2017) within the human *ASCL1* 1,304 bp region (Figure 5F). First, we tested the ability of an equivalent mouse *Ascl1* 1,043 bp region (Enh-*Ascl1*-luciferase) to respond to a strong activator such as Sox2 in NSCs (Figure 5G). We observed a robust transcriptional activation in luciferase-based reporter assays of cells transfected with Sox2-pCIG with respect to control pCIG, an activity probably mediated through the conserved putative Sox-binding sites (11 ± 2.4-fold relative to pCIG1; p = 0.0004; Figure 5G). However, when we tested Sox5 and/or Sox6 factors, we observed a lot of transcriptional variation in each experiment, with half of the cases showing a moderate increase in luciferase activity driven by Enh-*Ascl1* and the other half showing a reduction (Figure 5G). Thus, we concluded that the isolated 1 kb *Ascl1* enhancer was not consistently activated by Sox5 or Sox6 *in vitro*.

For that reason, we resorted to an *in vivo* full-genome assay. We used a Sox6 antibody successfully described on chromatin immunoprecipitation (ChIP) (Lee et al., 2017) and performed ChIP assays in rat adult hippocampus NSCs, using an acetylated Histone3 antibody as a positive control. By real-time qPCR (Figure 5H), we found that Sox6 antibody specifically immunoprecipitated a 214 bp *Ascl1* DNA fragment that comprises the three putative Sox-binding sites (0.0044 ± 0.0006 versus 0.0008 ± 0.0003 for IgG, expressed as percentage input; p = 0.0094; Figure 5H). In conclusion, SoxD factors activate *Ascl1* expression in adult hippocampus NSCs, and at least Sox6 may act directly through evolutionary conserved Sox-binding sites in a neural *Ascl1* enhancer.

### Sox5 loss hinders RGL activation in response to environmental enrichment

**Figure 6.**
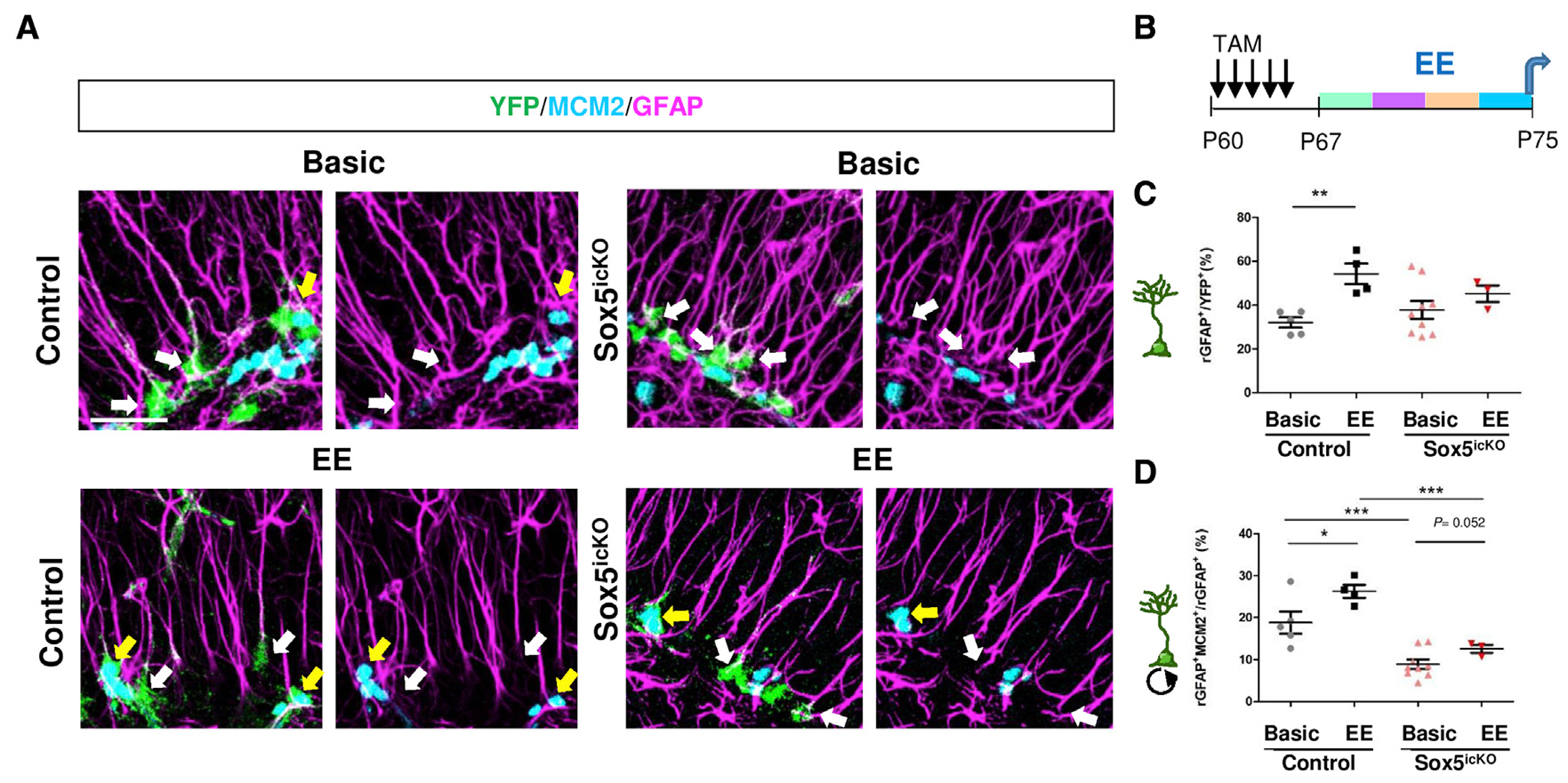
Sox5 loss hinders RGL activation in response to environmental enrichment (A) Confocal images showing YFP, MCM2, and GFAP at 14 dpi in control and in Sox5^icKO^ mice exposed to conventional (basic) or enriched (EE) environmental conditions. White arrows indicate YFP^+^ rGFAP^+^ MCM2^−^ cells and yellow arrows YFP^+^ rGFAP^+^ MCM2^+^ cells. (B) Experimental design for control and Sox5^icKO^ mice in which TAM i.p. injection was performed at P60 on 5 consecutive days, and after 7 days animals were exposed to an enriched environment for 8 days, changing objects every other day. (C) Quantitation of the number of radial GFAP in YFP^+^ cells in control and Sox5^icKO^ mice. (D) Quantitation of the number of rGFAP^+^ MCM2^+^ in rGFAP^+^ YFP^+^ cells. Results are shown as mean value ± SEM. *p < 0.05, **p < 0.01, and ***p < 0.001 by unpaired Student’s t test. Scale bar, 25 μm.

Adult neurogenesis is dynamically regulated by a variety of physiological and pathological factors such as physical exercise, task learning, environmental enrichment (EE), and seizures (Rolando and Taylor, 2014). However, it is not always clear whether changes in neurogenesis are due to alterations in RGL activation or to adaptations in proliferation and survival of IPCs and immature neurons. We decided to use 8 days of exposure to EE stimuli, including a running wheel, as potent physiological stimuli of adult neurogenesis. Using Control *Sox2-creER*^*T2*^/*Rosa26-YFP* mice injected for 5 days with TAM, we prepared groups of two males in basic cages under standard conditions (basic group) and groups of five males in enriched conditions, including larger cages with changing toys, tubes, running wheel, and bedding material (EE group; Figure 6B). At 15 dpi, we observed a clear increase in the number of YFP^+^ RGLs relative to the YFP^+^ population in EE mice in comparison with basic mice (54.3% ± 4.7% versus 32.1% ± 2.3%, respectively; p = 0.005; Figures 6A and 6C). Moreover, the percentage of activated RGLs (rGFAP^+^ MCM2^+^ cells) was higher in EE than in basic mice (26.3% ± 1.5% versus 18.8% ± 2.7%, respectively; p = 0.046; Figures 6A and 6D). Thus, we have shown that 8 days of EE promotes RGL activation in the adult hippocampus.

Then we analyzed whether Sox5^icko^ animals could similarly respond to EE cues. The relative number of RGLs in recombined YFP^+^ cells did not change in Sox5^icKO^ mice exposed either to the basic or the EE condition with respect to control mice (38.1% ± 11.11% versus 32.1% ± 4.7% in basic; 45.2% ± 5.3% versus 54.3% ± 8.0% in EE; Figures 6A and 6C). Regarding RGL activation, EE Sox5^icko^ mice did not reach the level of RGL activation observed in EE control mice (12.6% ± 0.9% versus 26.3% ± 1.5%, p = 0.001; Figures 6A and 6D). Nevertheless, EE Sox5^icko^ mice showed a tendency toward a 40% increase in RGLs activation with respect to Sox5^icko^ mice in basic cage conditions (12.6% ± 0.9% versus 8.9% ± 1.1%, p = 0.052; Figures 6A and 6D). Using a two-way ANOVA, we observed two significant effects: a genotype effect that accounts for 52.02% of the total variance (p < 0.0001) and an environment effect accounting for 11.56% of the total variance (p = 0.0084). In summary, our data indicate that although RGLs in Sox5^icko^ mice show some response to EE stimuli, enriched Sox5^icko^ mice failed to reach the level of RGL activation observed in enriched control mice.

## DISCUSSION

The potential to generate new neurons in spatially restricted brain areas during adulthood relies heavily on the regulation of the proliferative capacity of NSCs, a population predominantly in a state of reversible quiescence. Deciphering how RGLs, the resident adult NSC population, integrate physiological stimuli and local cues through their intrinsic transcriptional programs to abandon quiescence, and adjust the production of mature neurons, is essential to understand adult neurogenesis. Here, we show that SoxD proteins (Sox5 and Sox6) are required for RGL activation in the hippocampal neurogenic niche, that quiescent signals such as BMP4 modulate their transcription, and that Sox5 mutants do not exhibit the full RGL activation response to physiological neurogenic stimuli such as EE. Furthermore, Sox5 and Sox6 activate the expression of the proneural *Ascl1* gene, which encodes a factor critical in adult RGL activation (Andersen et al., 2014). In summary, *SoxD* genes emerge as key factors for the control of adult neurogenesis.

To begin with, we have established that SoxD factors are crucial in adult RGLs to transit from a quiescent to an activated proliferative state. Loss of SoxD factors in adult RGLs, after just 7 days, reduced by more than half the ability of quiescent RGLs to engage in cell cycle progression. Conversely, increasing Sox5 levels hinders the cells ability to enter quiescence when they receive such pro-quiescent signals as BMP4. Moreover, the defect in RGL proliferation caused by SoxD loss is robust, as it lasts at least 1 month after gene deletion. We reinforced that idea by demonstrating that the RGLs that have recently undergone a round of cell division, upon losing Sox5, are unable to re-enter the cell cycle and thus remain in a quiescent state. This could also indicate that losing Sox5 increases the number of resting RGLs, which are those RGLs in a shallow quiescent state, more prone to engage in cell division (Harris et al., 2021; Urbán et al., 2016). However, without Sox5, those resting RGLs could not engage in cell division.

The importance of the fine control of the activation of RGLs is stressed by the fact that only 7 days after the loss of SoxD factors, there is an immediate impact on neurogenesis, observed by a reduced number in immature neurons, which do not seem to be compensated by IPC overproliferation. Furthermore, the effect is maintained for at least 1 month, when there is still a sustained reduction in new neurons in both Sox5^icKO^ and Sox6^icKO^ mice. In any case, SoxD function in RGLs does not appear to be related to cell-fate decisions, as RGLs in Sox5^icKO^ and Sox6^icKO^ mice, incapable of entering the cell cycle, do not opt for direct differentiation into astrocytes.

The SoxD family members Sox5 and Sox6 are closely related DNA-binding proteins, sharing 87% identity and acting as homodimers and heterodimers (Lefebvre, 2010). They share several common target genes (Lee et al., 2017; Liu et al., 2017) and have redundant functions when they are co-expressed in the same cell type (Stolt et al., 2006). Now we have shown that Sox5 and Sox6 share patterns of expression in the adult neurogenic niche and that both are required for RGL activation and for adult neurogenesis.

Although significant progress has been made in describing extrinsic and intrinsic cues that control RGL behavior, relatively few advances have been made in understanding how cells integrate that information. We have now shown how quiescence-promoting signals such as BMP4 (Martynoga et al., 2013; Mira et al., 2010), at least in NSCs *in vitro*, downregulate *Sox5* and *Sox6*. Ascl1 protein levels are also strongly reduced in BMP4-treated quiescent NSCs (Blomfield et al., 2019), and both Id4 transcription factor and E3 ubiquitin ligase Huwe1 (Urbán et al., 2016) regulate Ascl1 at the protein level. However, the mechanism controlling *Ascl1* transcription *in vivo* in RGLs is not fully clear, although it involves repression by Notch signaling (Imayoshi et al., 2013; Sueda et al., 2019). Now we have unveiled another layer of Ascl1 regulation showing that Sox5 and Sox6 modulate Ascl1 expression in RGLs *in vivo* and that at least Sox6 may control *Ascl1* transcription through an evolutionary conserved and transcriptionally active neural enhancer in *Ascl1* locus.

Finally, a particularly important feature of hippocampal neurogenesis is its regulation by a variety of physiological stimuli such as EE (Rolando and Taylor, 2014). In the case of EE, acute EE stimulus could induce general cell proliferation (Chandler et al., 2020; Steiner et al., 2008), and long-term EE affects only cell survival, not proliferation (Kempermann et al., 1997; van Praag et al., 1999). We have now demonstrated that qRGLs respond to an 8 day EE paradigm entering in an active proliferative state. More important, Sox5 loss hinders the RGL response to EE physiological cues.

In conclusion, we have demonstrated how SoxD family members are required for RGL activation modulating Ascl1 expression and for the generation of new neurons in the adult hippocampus. Understanding the molecular mechanisms controlling adult NSC activation will open new approaches for regenerative medicine and treatment of brain disorders. Moreover, given the fact that *SOX5* and *SOX6* heterozygous inactivating variants cause neurodevelopmental diseases in humans (Lamb-Shaffer and Tolchin-Le Caignec syndromes, respectively), our findings will also shed light on the neural alterations present in those SOXopathies (Angelozzi and Lefebvre, 2019).

### Limitations to the study

As we achieved only partial loss of Sox5 or Sox6 in our conditional mice, we are aware of the very likely dilution of the observed phenotype due to inefficient recombination of *SoxD* genes.

As we did not have a good Sox5 antibody for immunoprecipitation, we could not establish Sox5 direct binding to the site occupied by Sox6 in the *Ascl1* enhancer.

There is a limitation in the statistical interpretation of EE results, as there is an imbalance between Sox5 mutants in control (n = 9) versus EE (n = 3) conditions. Thus, the tendency observed in Sox5 mutants toward 40% RGL activation upon EE could be of statistical significance (p = 0.052).

## STAR★METHODS

### RESOURCE AVAILABILITY

#### Lead contact

Further information and requests for resources and reagents should be directed to and will be fulfilled by the lead contact, Aixa V. Morales (aixamorales@cajal.csic.es).

#### Materials availability

Transgenic lines and plasmids used in this study can be obtained upon request.

#### Data and code availability

- Microscopy, cell counting, RT-qPCR, and cell transduction data reported in this paper will be shared by the lead contact upon request.
- This paper does not report original code.
- Any additional information required to reanalyze the data reported in this paper is available from the lead contact upon request.

### EXPERIMENTAL MODEL AND SUBJECT DETAILS

#### Mice

All experiments were performed in 2- to 4-months-old C57BL/6J mice of both genders. Animal procedures were carried out in accordance with the guidelines of European Union (2010/63/UE) and Spanish legislation (53/2013, BOE no. 1337). Mice were housed with a standard control of a 12 h light/dark cycle and maintained in the animal facility at Cajal Institute.

*Sox2-EGFP* mice, in which 5.5 kb brain-specific *Sox2* promoter drives EGFP expression, were obtained from R. Gage laboratory (Suh et al., 2007). *Sox5*^*fl/fl*^ mice, in which coding exon 5 of *Sox5* gene is flanked by loxP sites (Dy et al., 2008), or *Sox6*^*fl/fl*^ mice, in which coding exon 2 of *Sox6* gene is flanked by loxP sites (Dumitriu et al., 2006) were used. They were bred with *Sox2-creER*^*T2*^ mice (Favaro et al., 2009) and with Rosa26-floxed stop-YFP reporter mice (Srinivas et al., 2001) to generate control animals (Control): *Sox2-creER*^*T2*^/*YFP^+^, Sox2-creER*^*T2*^/*Sox5*^*fl*/+^/*YFP*^+^ or *Sox2-creER*^*T2*^/*Sox6*^*fl/+*^/*YFP*^+^ and mutant mice: *Sox2-creER*^*T2*^/*Sox5*^*fl/fl*^/*YFP*^+^ (Sox5^icKO^), *Sox2-creER*^*T2*^/*Sox6*^*fl/fl*^/*YFP*^+^ (Sox6^icKO^) or double mutant *Sox2-creER*^*T2*^/*Sox5*^*fl/fl*^/*Sox6*^*fl/fl*^/*YFP*^+^ (Sox5^icKO^/Sox6^icKO^).

#### Primary cell cultures

Adult hippocampal NSCs were cultured as previously described (Nieto-Estevez et al., 2016). Briefly, mice (6- to 8-weeks old of both genders) were euthanized with CO_2_, their brains were isolated and the hippocampus were dissected, cut up into pieces and digested with Papain [0.66 mg/ml papain (Worthington) + 0.2mg/ml cysteine (Sigma) + 0.2 mg/ml EDTA (Merck) + Hank’s buffer (Thermo Fisher)] for 20min at 37°C. After mechanical dissociation and washes with DMEM F12 (Thermo Fisher) to stop the reaction and washes with Hank’s, the disaggregated cell suspension was plated into MW12 plates with basal media [DMEM F12 + 1X N2 supplement (100X; Thermo Fisher) + 1X B27 supplement (50X; Thermo Fisher)], 20 ng/ml EGF (100 ng/ μl; PeproTech) and 20 ng/ml FGF-2 (100 ng/ μl; PeproTech). Cells were incubated at 37°C and 5% CO_2_. Normally, one single brain was used to prepare the culture and 4 wells of MW12 per brain were used. For hippocampal floating neurospheres, 20 ng/ml EGF and 20ng/ml FGF-2 were daily added and were passaged by mechanical procedures and used from passage 3 until passage 25 for different cell treatments.

For the ChIP assays we used rat Adult Hippocampal Neural Stem and Progenitor Cells that were maintained as neurospheres in DMEM F-12 (Gibco, Thermo Fisher) adding N2 Supplement (1x) with 20 ng/ml of FGF-2 (PeproTech).

### METHODS DETAILS

#### *In vivo* treatments

For activation of the creER^T2^ recombinase, 2- to 3-months-old animals (both Control and the indicated inducible mutant mice) were administered intraperitoneally (ip) a dose of 5 mg/40gr body weight of TAM (20 mg/ml in corn oil; Sigma) for 5 consecutive days. Animals were perfused 7, 14 or 30 days post TAM injection (dpi). To examine slowly dividing RGLs, mice received 5 BrdU injections in a three days period and were sacrificed 30 days later.

#### Tissue preparation and immunofluorescence

Animals were transcardially perfused with saline followed by 4% paraformaldehyde (PFA). Brains were postfixed with 4%PFA for 3 h at 4°C, embedded in 30% agarose/sucrose (w/v) and sectioned coronally at 50μm using a vibratome.

For immunostaining, vibratome floating brain sections were permeabilized with 1% Triton X-100 in 0.1M Phosphate Buffer (PB) for 30 min and blocked with 10% Fetal Bovine Serum (FBS) and 0.25% Triton X-100 in 0.1M PB for 2 h at room temperature with rocking. For fixed cells, permeabilization and blocking were performed with 10% FBS in 0.25% Triton X-100 in 0.1M PB for 1 h at room temperature. Primary antibodies were prepared in incubation buffer (1% FBS, 0.25% Triton X-100 in 0.1M PB) and incubated with sections or cells overnight at 4°C. Following 3 washes, with washing buffer (0.1% Triton X-100 in 0.1M PB), sections or cells were incubated with secondary antibodies (1:1000) in incubation buffer for 2 h at room temperature. After three washes, sections or cells were incubated with bisbenzimide (1:100 in 0.1M PB) for 2 min at room temperature, and mounted using Fluoromount-G (Thermo Fisher).

To detect MCM2 protein, antigen retrieval was carried out by incubating sections in 0.15M sodium citrate at 80°C for 30min. To detect BrdU incorporation, DNA was denatured by incubating sections or cells with 2N HCL for 25min at room temperature, followed by 0.15M boric acid neutralization for 20min. When MCM2 or BrdU staining was performed combined with EGFP immunostaining, the latter (including primary and secondary antibodies) was done prior to citrate or acid treatments.

#### Primary cell culture nucleofection

Neurospheres were dissociated and grown as adherent cells in poly-L-ornithine (1.5 mg/ml; Sigma) and fibronectin (1 mg/ml; Invitrogen) coverslides. To induce quiescence, cells were plated at a density of 80,000 cells/MW24 well in basal media with 20 ng/ml FGF2 and after 24 h, media was replaced with basal media with 20 ng/ml FGF2 alone or in combination with with 30 ng/mL recombinant mouse BMP4 (100 ng/ μl; PeproTech). Cells were cultured for 72 h and then fixed with 4% PFA (Merck) on ice for 20min. Brdu (10 mg/ml; Roche) was added to the media for 1 h before fixation.

Sox5 coding sequence inserted into a pCIG vector containing EGFP (pCIG-Sox5) has been described before (Quiroga et al., 2015). As a control, empty pCIG vector (pCIG-EGFP) was used. 2.5 × 10^6^ cells from dissociated neurospheres derived from 6-weeks old wild type mice hippocampus were nucleofected with DNA plasmids at a concentration of 2.5 μg/μl using the P3 Primary Cell 4D-Nucleofector^™^ X Kit (Lonza, V4XP-3012) and the program #113, based on the manufacturer’s instructions. After nucleofected, cells were cultured at a density of 80,000-150,000 cells/MW24 well in adherent conditions as described above.

DNA overexpression assays in Figure 5C were performed by Effectene Transfection Reagent (Qiagen) in adherent adult hippocampal NSCs in MW6 plates (280,000 cells/well) coated with polyornithine and fibronectin. Following Qiagen instructions, 3 μg of DNA plasmid was first mixed with Enhancer solution in a buffer that provides optimal salt conditions for efficient DNA condensation during 5 min. Effectene Reagent was then added and incubated for 10 min at RT. The complexes were mixed with neurospheres growth medium and added directly to adherent NSCs during 5 h. After removing the transfecting reagent cells were incubated for 24 h and then collected for total RNA extraction.

#### Lentiviral transduction

Adult mouse hippocampal NSCs were plated at a concentration of 60,000 cells/well in MW6 and 24 h after were transduced with lentiviral particles (5 units of multiplicity of infection; MOI) expressing three different Sox5 mouse shRNA (A to C; Locus ID, 20678; Cat# TL502111V; Origene) or one standard control scrambled shRNA (Origene). All the constructs express EGFP as a reporter. After adding lentiviral particles, plates were centrifuged for 1.5 h at 1050 rpm at RT and then viral transduction took place for 3–4 days. Cells were collected and total RNA was extracted.

#### Luciferase reporter assay

An evolutionarily conserved region of 1304 bp, located 6.6 kb upstream of the human *ASCL1* coding region, was detected (hg19 chr12:103,345,375-103,346,678; ATG in 103,352,023; TSS 103351452). An equivalent 1043 bp fragment of the mouse *Ascl1* genomic locus (positions −8479 to −7446 from coding sequence) was cloned upstream a minimal CMV promoter-luciferase reporter gene (Vector Builder Company; Enh-*Ascl1*-Luciferase). Adherent NSCS cultured in MW12 plates (140,000 cells/well) were transfected using Effectene Transfection Reagent (Qiagen) with 0.5 μg Enh-*Ascl1*-Luciferase and a combination of 1 μg of Sox5 and/or Sox6 or Sox2 expressing constructs in vector pCIG, in combination with 50 ng of SV40-Renilla and 10 ng and CMV-Renilla constructs for normalization. After 24 h, cells were harvest and luciferase activities were measured by Dual Luciferase Reporter Assay System (Promega).

#### RNA extraction, cDNA synthesis and real-time quantitative PCR

Cultured hippocampal NSCs (100,000cells/MW24 well) grown in adherent conditions with FGF2 or with FGF2 plus BMP4 were collected after three days of treatment and stored at −80°C. Total RNA was extracted using the QuickGene RNA tissue kit S (Kurabo) and then treated with DNAse. cDNA was synthesized with SuperScriptTM IV First-Stand Synthesis System and random hexamers (Invitrogen). Gene expression levels were measured using TaqMan Gene expression assays (Applied Biosystems) and real-time quantitative PCR (qPCR) was carried out in a 7500 PCR System using TaqMan Fast Advanced Master Mix (Applied Biosystems). Gene expression was measured relative to endogenous controls *Pgk1* and *Gapdh*, and normalized to the expression of the Control sample in each group using the 2^^^(−ΔΔCt) method, as indicated in the corresponding figure. At least three independent experiments were performed for each condition and samples were run in triplicates.

#### Chromatin immunoprecipitation (ChIP) assay

6 × 10^6^ NSCs from the adult rat hippocampus (for each ChIP reaction) were cross-linked in medium containing 1% formaldehyde for 10 min at 37°C and neutralized with 0.137 M glycine for 5 min at room temperature. Next, cells were scraped off and rinsed twice with 10 ml of cold PBS containing protease inhibitors. Cells were pelleted by centrifugation for 4 min at 5000 rpm at 4°C. Cell pellets were suspended in SDS Lysis buffer (1% SDS, 1 mM EDTA, 50 mM Tris pH 8.1) and incubated for 10 min at 4°C. Lysates were sonicated using a Bioruptor Sonicator (Diagenode, BioRuptorTM UCD-200) and cellular debris was removed by spinning at 13,000 rpm for 10 min at 4°C. The supernatant fraction was diluted 5-fold in ChIP Dilution Buffer and chromatin solution was incubated overnight using Anti-acetyl-Histone H3, Anti-Sox6 and Anti-rabbit IgG after 1 h of preclearing at 4°C. The immune-complexes were incubated with Protein A agarose/Salmon Sperm DNA (Millipore), washed and eluted in elution buffer (1% SDS/0.1 M NaHCO3). Next, the cross-linking was reversed at 65°C overnight with gentle shaking. The DNA was purified by phenol–chloroform extraction followed by ethanol precipitation and was recovered in 20 μl of DNAse free water.

ChIP quantification was performed by quantitative PCR using SYBR PremixEX Taq (2x; Takara) amplifying a 214 bp fragment of the rat *Ascl1* gene. ChIP-qPCR data was analyzed by % Input method based on Ct value normalized by the amount of chromatin input. The value for each sample is calculated as follows: % Input = 100*2^^^ [Adjusted input - Ct (IP)]. Primer set details are in key resources table.

#### Environmental enrichment

We used an EE protocol involving classical toys and objects, and a running wheel (Kempermann et al., 1997). Control mice were housed in groups of two in transparent Plexiglas cages (20 × 22 × 20 cm) under standard laboratory conditions. Mice subjected to EE were housed in groups of five animals in large transparent Polycarbonate cages (55 × 33 × 20 cm, Plexx Ref. 13,005). All enriched cages were equipped with different types of running wheels; different bedding material (newspapers, sheets of paper, sawdust and cloth); toys used included plastic non-edible ones as hard plastic toys, small plastic animals, gumabones (Plexx Ref. 13,110), tips, pet balls, a transparent polycarbonate mouse igloo (Plexx Ref. 13,100), polycarbonate rodent tunnels (Plexx Ref. 13,102) and others. Every other day, a set of 10–20 different toys and new bedding material was introduced into the cages and in general, the complexity of the cage environment was changed completely.

### QUANTIFICATION AND STATISTICAL ANALYSIS

#### Microscopic analysis and cell counting

All images were taken with a direct SP5 confocal microscope (Leica). Images of both left and right dorsal DG sections (−0.82 mm to −4.16 mm from bregma) were captured with a z-step of 2 μm through at least 20 μm of each 50 μm sections. Labeled cells were counted in the SGZ of every ninth of 50 μm DG sections. In wild type and *Sox2-EGFP* mice the analysis was done counting the number of cells (at least 300 cells/marker) that expressed a cell-type specific maker in the population of Sox5^+^ or Sox6^+^ cells and number of Sox5^+^ cells among SGZ cells expressing a certain cell-type marker. In Control, single mutants Sox5^icKO^ or Sox6^icKO^ and in double mutant mice we counted recombined YFP^+^ cells that were positive for the indicated marker. In those cases, 3 to 6 sections from at least 3 to 7 mice and a minimum of 100 cells for each animal were analyzed. Counting was performed manually and blind using LAS X (Leica) software. In order to measure Sox5 immunofluorescence intensity in the RGL population, the nucleus of each RGL (characterized by YFP recombination with a radial process) was manually outlined according to bisbenzimide staining, and the average intensity of Sox5 immunostaining was measured using FIJI software.

For the experiments with adherent adult hippocampal NSCs grown in FGF2 or BMP4, six random regions in each of, at least, three coverslips for each experimental situation were photographed with a z-step of 2 μm by 40X objective. For the experiments of TAM-induced and nucleofected cells, at least 100 recombined or transfected cells were photographed with the same conditions. In all the cultures, three independent experiments were performed for each condition. For *in vitro* measurement of immunofluorescence intensity, an ImageJ macro was designed to first identify all the nuclei and then measure the average intensity of the selected channel for the area of each nucleus automatically, using ImageJ software. To quantify proliferation in cultured cells, ImageJ macros were designed to measure the percentage of proliferating cells (BrdU^+^ or Ki67^+^ cells) among identified nucleus using ImageJ software. Differentiation markers in cultured cells after TAM administration were quantified manually. At least 100 EGFP positive cells were evaluated among EGFP^+^ cells.

#### Statistical analysis

The appropriate sample size (N) was determined based on similar published data from other groups (Andersen et al., 2014; Bonzano et al., 2018), using a minimum of 3 mice per condition for *in vivo* experiments, and a minimum of biological triplicate for *in vitro* experiments. Statistical analysis and graphs were conducted with GraphPad Prism version 5 software using different tests with a significance level of at least *p* < 0.05. Thus, two-tailed unpaired Student’s t test were used for most of statistical comparisons of two conditions for *in vivo* experiments; paired t test for *in vitro* experiments where control and treatment conditions for each biological replicate were performed in parallel, in Figures 3; 5 and S5. Finally, two-way ANOVA was used in Figure 6 for four conditions. Statistical details were included in each figure and figure legend [number of experiments (N), number of cells (n) and statistical test]. Data are presented as mean ± SEM. Significance is stated as follows: p < 0.05 (*), p < 0.01 (**), p < 0.001 (***), confidence intervals of 95%.

## Supplementary Material

1

## Figures and Tables

**Table:** KEY RESOURCES TABLE

REAGENT or RESOURCE	SOURCE	IDENTIFIER
Antibodies		
Rabbit anti-acetyl-Histone H3	Millipore	Cat#06-599; RRID: AB_2115283
Mouse anti-Ascl1	BD Biosciences	Cat#556604; RRID: AB_396479
Rat anti-BrdU	Abcam	Cat#ab6326; RRID: AB_10783500
Goat anti-DCX	Santa Cruz Biotechnology	Cat#sc-8066; RRID: AB_2088491
Rabbit anti-GFAP	Dako	Cat#Z0334; RRID: AB_2811722
Rat anti-GFP	Nacalai Tesque	Cat#GF090R; RRID: AB_10013361
Rabbit anti-IgG	Abcam	Cat#ab3741; RRID: AB_1951970
Mouse anti-Ki67	BD Biosciences	Cat#550609; RRID: AB_393778
Mouse anti-MCM2	BD Biosciences	Cat#610700;RRID: AB_2141952
Mouse anti-PSA NCAM	Hybridoma Bank	Cat#5A5; RRID: AB_528392
Goat anti-Sox2	R&D (Vitro)	Cat#AF2018; RRID: AB_355110
Rabbit anti-Sox5	AV Morales (I. Cajal); (Quiroga et al., 2015)	N/A
Guinea pig anti-Sox6	M. Wegner (Erlangen-Nürnberg); (Stolt et al., 2006)	N/A
Rabbit anti-Sox6 (ChIp)	Abcam	Cat#Ab30455; RRID: AB_1143031
Rabbit anti-S100	Abcam	Cat#Ab868; RRID:AB_777793
Rabbit anti-S100β	Merck	Cat#S2532
Rabbit anti-Tbr2	Abcam	Cat#Ab23345; RRID: AB_778267
Chicken anti- βIII Tubulin	Abcam	Cat#Ab41489;RRID: AB_727049
Donkey anti-rabbit IgG-Alexa Fluor 488	Invitrogen	Cat#A-21206; RRID:AB_2535792
Donkey anti-rat IgG (H+L)-Alexa Fluor 488	Invitrogen	Cat#A21208; RRID:AB_141709
Goat anti-guinea pig IgG- Alexa Fluor 488	Jackson Immunoresearch	Cat#106-545-003; RRID: AB_2337438
Goat anti-mouse IgG1- Alexa Fluor 546	Invitrogen	Cat#A21123; RRID:AB_141592
Donkey anti-goat IgG (H+L)-Alexa Fluor 594	Invitrogen	Cat#A11058; RRID:AB_2534105
Donkey anti-guinea pig IgG (H+L)- Alexa Fluor 594	Jackson Immunoresearch	Cat#706-585-148; RRID: AB_2340474
Donkey anti-mouse IgG- Alexa Fluor 549	Invitrogen	Cat#A21203; RRID: AB_141633
Goat anti-chicken IgG (H+L)- Alexa Fluor 594	Invitrogen	Cat#A11042; RRID: AB_2534099
Donkey anti-goat IgG- Alexa Fluor 647	Abcam	Cat#Ab150131; RRID: AB_2732857
Donkey anti-mouse IgG- Alexa Fluor 647	Invitrogen	Cat#A-31571; RRID: AB_162542
Bacterial and virus strains		
Sox5 Mouse shRNA Lentiviral Particles (Locus ID 20678)	Origene	Cat#TL502111V
Chemicals, peptides, and recombinant proteins		
Tamoxifen	Sigma	Cat#T5648-1G
Bisbenzimide	Sigma	Cat#016M41
Fluoromount-G	Fisher Scientific	Cat#00-4958-02
4-hydroxytamoxifen	Sigma-Aldrich	Cat#H7904
Recombinant human-Bone Morphogenetic Protein-4	Peprotech	Cat#120-05ET
Recombinant human-Fibroblast Growth Factor-2	Peprotech	Cat#100-18B
Recombinant human-Epidermal Growht Factor	Peprotech	Cat#100-15
N2 Supplement	Fisher Scientific	Cat#17502048
B27 supplement	Fisher Scientific	Cat#17504044
DMEM/F12	Gibco	Cat#42400-010
HBSS	Fisher Scientific	Cat#24020117
Papain	Worthington	Cat#33H14332L
Cysteine	Sigma Aldrich	Cat#C7352
Poly-L-ornithin	Sigma Aldrich	Cat#P3655
Fibronectin	Invitrogen	Cat#33010-018
Insulin	Sigma Aldrich	Cat#16634
Corn Oil	Sigma Aldrich	Cat#C8267
Critical commercial assays		
Effectene Transfection Reagent	Quiagen	Cat#301425
P3 Primary Cell 4D-NucleofectorTM X Kit	Lonza	Cat#V4XP-3012
Dual Luciferase Reporter Assay System	Promega	Cat#E1910
QuickGene RNA tissue Kit S	Kurabo	Cat#RT-S2
SuperScript^™^ IV First-Stand Synthesis System	Invitrogen	Cat#18091050
TaqMan Fast Advanced Master Mix	Applied Biosystems	Cat#A44360
Experimental models: Organisms/strains		
Mouse: C57Bl/6	Charles River Laboratories	N/A
Mouse: *Sox2:CreER^T2^*	S. Nicolis (U. Milano-Biccoca); (Favaro et al., 2009)	http://www.informatics.jax.org/allele/MGI:4397776
Mouse: *Sox2-EGFP*	F. Gage (Salk Institute); (Suh et al.,2007)	N/A
Mouse: *Sox5^flox/flox^*	V. Lefebvre (Philadelphia); (Dy et al., 2008)	http://www.informatics.jax.org/allele/MGI:3799354
Mouse: *Sox6 ^flox/flox^*	V. Lefebvre (Philadelphia); (Dumitriu et al., 2006)	http://www.informatics.jax.org/allele/MGI:3641205
Mouse: *Rosa26^lox-stop-lox-YFP^*	The Jackson Laboratory	https://www.jax.org/strain/006148
Oligonucleotides		
*Ascl1* Probe	Applied Biosystems	Mm03058063_m1
*Bmpr1a* Probe	Applied Biosystems	Mm00477650_m1
*Bmpr1b* Probe	Applied Biosystems	Mm03023971_m1
*Gapdh* Probe	Applied Biosystems	Mm99999915_g1
*Id2* Probe	Applied Biosystems	Mm00711781_m1
*Id4* Probe	Applied Biosystems	Mm00499701_m1
*Pgk1* Probe	Applied Biosystems	Mm00435617_m1
*Sox5* Probe	Applied Biosystems	Mm01264584_m1
*Sox6* Probe	Applied Biosystems	Mm00488393_m1
Primer sequences: *Ascl1* Fw: CGCTCCTGTCGCTGAGGTGTTTC	Sigma	Forward Primer
Primer sequences: *Ascl1* Rv: GCTTCCCCCTCACAATCACAGG	Sigma	Reverse Primer
Recombinant DNA		
Plasmid pCIG-Sox5	AV Morales (I. Cajal); (Quiroga et al., 2015)	N/A
Plasmid pCIG-Sox6	AV Morales (I. Cajal); (Quiroga et al., 2015)	N/A
Plasmid pCIG-Sox2	J. Muhr (Karolinska); (Kurtsdotter et al., 2017)	N/A
Plasmid pRP-Enh-*Ascl1*-Luciferase	Vector Builder Company	ID: VB210601-1396nca
Software and algorithms		
ECR Browser	I. Ocharenko (National Library of Medicine)	https://ecrbrowser.dcode.org/; RRID:SCR_001052
FIJI Software	ImageJ NIH	https://imagej.net/software/fiji/; RRID:SCR_002285
GraphPad Prism v.5	GraphPad Software, Inc.	https://www.graphpad.com; RRID:SCR_002798
ImageJ2	ImageJ NIH	https://imagej.net; RRID:SCR_003070
Leica Application Suite X	Leica	https://www.leica-microsystems.com; RRID:SCR_013673
MULAN	I. Ocharenko (National Library of Medicine)	https://mulan.dcode.org/
VISTA	Join Genome Institute (USA)	https://genome.lbl.gov/vista/index.shtml; RRID:SCR_018707

## Highlights

- Neural stem cell transition from quiescence to activation requires Sox5 and Sox6
- Loss of Sox5 or Sox6 decreases adult neurogenesis in the dentate gyrus
- Transcription of neural stem cell activator *Ascl1* is modulated by Sox5 and Sox6
- Environmental enrichment boosts stem cell activation, which is hampered by *Sox5* loss

## INCLUSION AND DIVERSITY

We worked to ensure sex balance in the selection of non-human subjects.
