# Supplementary material for: SoxD genes are required for adult neural stem cell activation
Source: Cell Rep. Author manuscript; Available in PMC 2025 Jan 31. (PMC11783645; doi:10.1016/j.celrep.2022.110313)
Supplement: 1 [file NIHMS2018114-supplement-1.pdf]

Cell Reports, Volume *38*

Supplemental information

SoxD genes are required for adult neural stem cell activation

Lingling Li, Cristina Medina-Menéndez, Laura García-Corzo, Carmen M. Córdoba-Beldad, Alejandra C. Quiroga, Elena Calleja Barca, Valeriya Zinchuk, Sara Muñoz-López, Pilar Rodríguez-Martín, Maria Ciorraga, Inés Colmena, Silvia Fernández, Carlos Vicario, Silvia K. Nicolis, Véronique Lefebvre, Helena Mira, and Aixa V. Morales

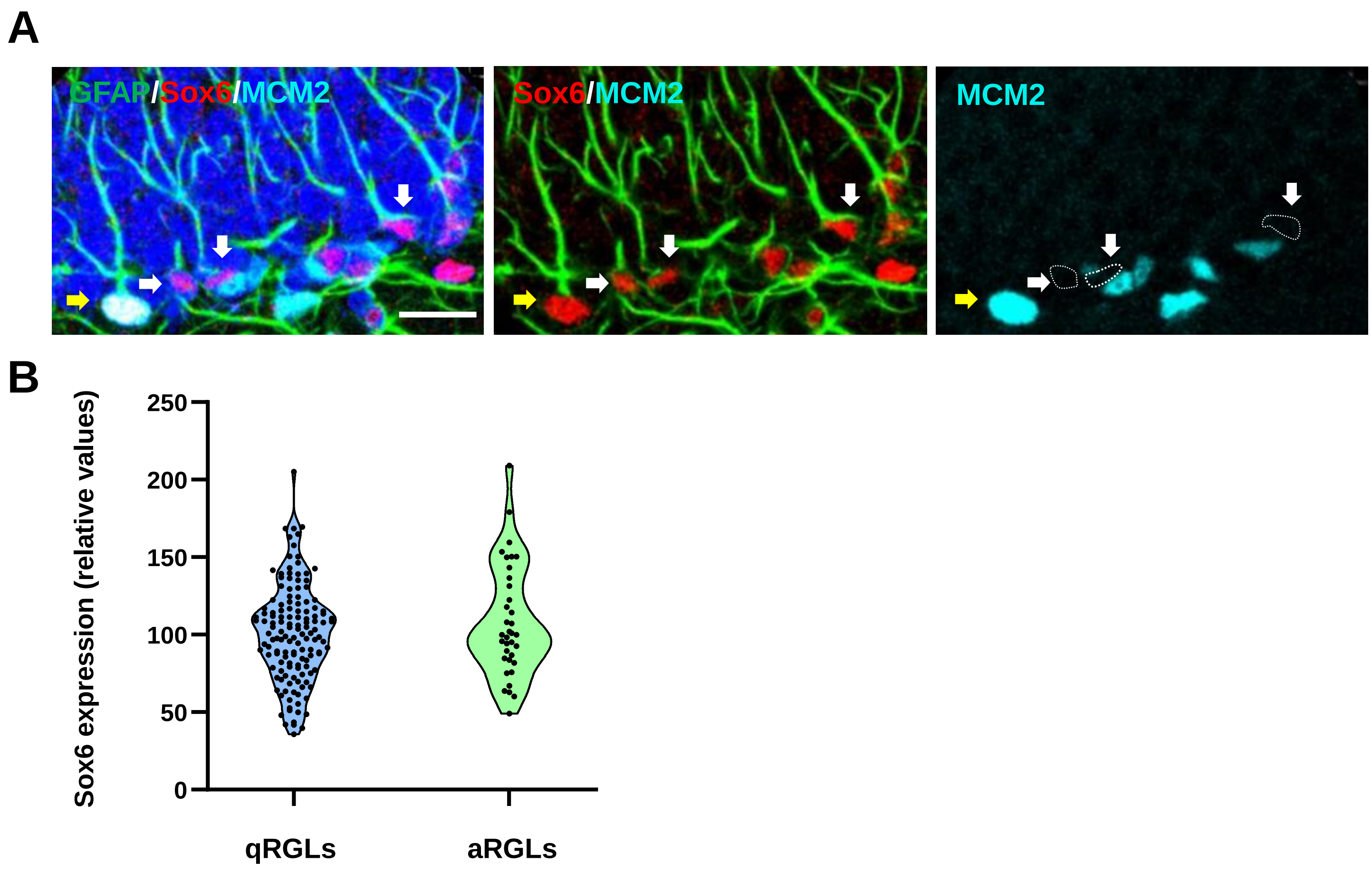

Figure S1. Sox6 is similarly expressed in active and in quiescence RGLs in the adult SGZ. Related to Figure 1. (**A**) Confocal images showing RGLs in the SGZ of 2 month-old mice using immunohistochemistry for GFAP, MCM2 and Sox6. White arrows show rGFAP$^+$Sox6$^+$MCM2$^-$ cells and yellow arrows rGFAP$^+$Sox6$^+$MCM2$^+$ cells. (**B**) Violin plot representing the intensity of Sox6 inmunofluorescence in GFAP$^+$MCM2$^-$ quiescent RGLs (qRGLs, n = 135) and in GFAP$^+$MCM2$^+$ active RGLs (aRGLs, n = 36), relative to Sox6 expression levels in qRGLs. Scale bar represents 15 μm.

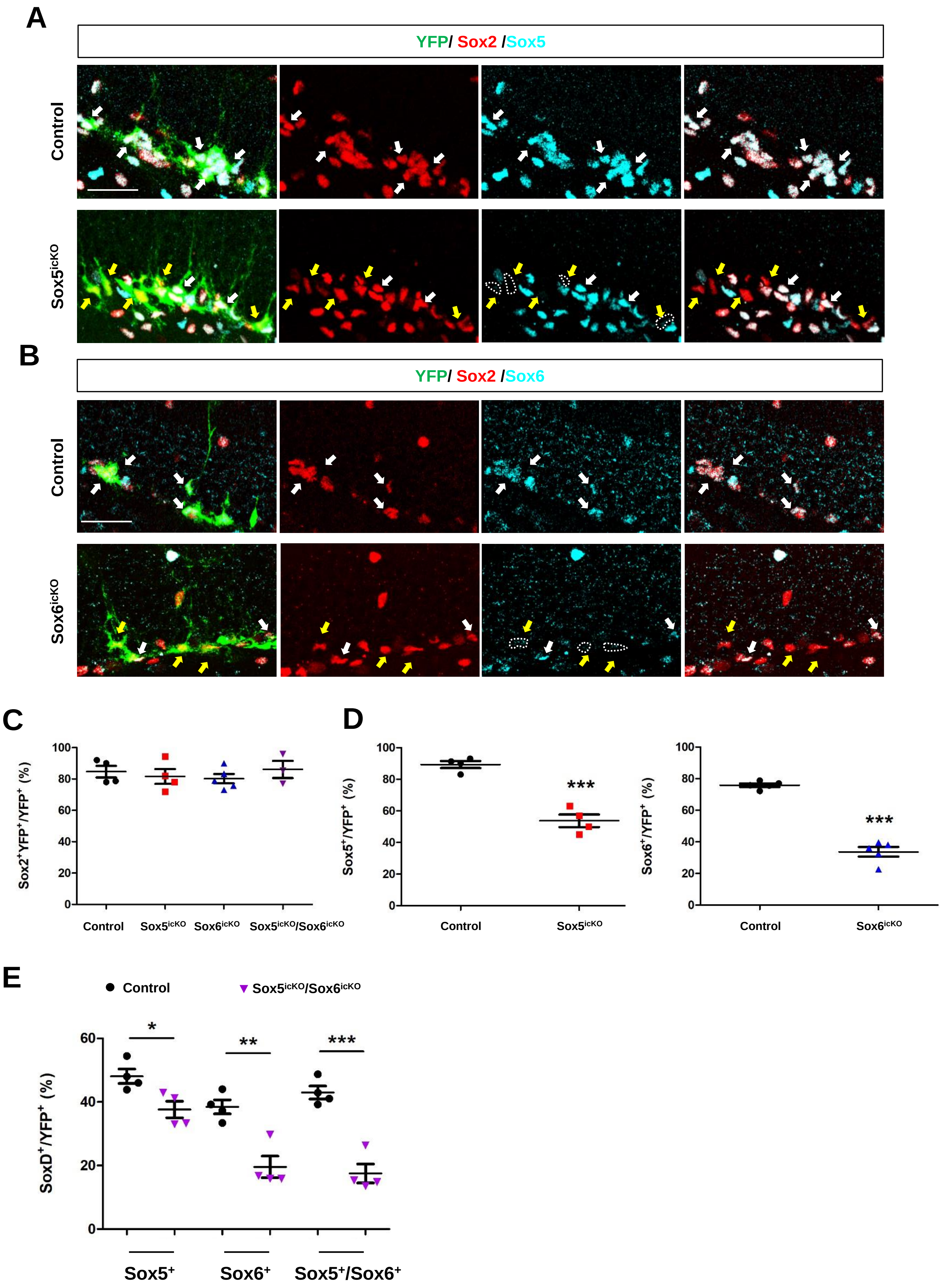

A
YFP/ Sox2 /Sox5
Control
Sox5icKO
B
YFP/ Sox2 /Sox6
Control
Sox6icKO
C
Sox2+YFP+/YFP+ (%)
100
80
60
40
20
0
Control
Sox5icKO
Sox6icKO
Sox5icKO/Sox6icKO
D
Sox5+/YFP+ (%)
***
Control
Sox5icKO
Sox6+/YFP+ (%)
***
Control
Sox6icKO
E
Control
Sox5icKO/Sox6icKO
SoxD+/YFP+ (%)
60
40
20
0
*
**
***
Sox5+
Sox6+
Sox5+/Sox6+

Figure S2. Sox5 and Sox6 expression are efficiently removed upon TAM-induced cre-recombination. Related to Figure 2. (A, B) Confocal images of the SGZ showing YFP, Sox2 and Sox5 or Sox6 inmunostaining in Control, $Sox5^{icKO}$ and $Sox6^{icKO}$ mice 7 days upon TAM-induced recombination. White arrows indicate YFP^+ $Sox2^+$ $Sox5^+$ or YFP^+ $Sox2^+$ $Sox6^+$ cells, and yellow arrows YFP^+ $Sox2^+$ $Sox5^-$ and YFP^+ $Sox2^+$ $Sox6^-$ cells. **(C)** Quantitation of the number of $Sox2^+$ RGLs and progenitors in recombined YFP^+ cells in the indicated mice. **(D)** Quantitation of the number of $Sox5^+$ or $Sox6^+$ cells among YFP^+ population in the SGZ of the indicated mice. **(E)** Quantitation of the number of $Sox5^+$, $Sox6^+$ or double $Sox5^+/Sox6^+$ cells among YFP^+ population in the SGZ of control and double $Sox5^{icKO}/Sox6^{icKO}$ mice. From this data we can calculate that in recombined YFP^+ cells in double $Sox5^{icKO}/Sox6^{icKO}$ mice: 17.5± 0.5 % of cells are $Sox5^+/Sox6^+$; 20.1± 2.2% are $Sox5^+/Sox6^-$; 2.1± 0.5% are $Sox5^-/Sox6^+$; and we estimate that the remaining population of 3.3± 2.7% will be $Sox5^-/Sox6^-$ (having as a reference a total of 43.0± 2.1 % $Sox5^+/Sox6^+$ cells in Control mice). Data represents mean values ± SEM. *** $P < 0.001$according to unpaired Student's t-test. Scale bar, 30 µm.

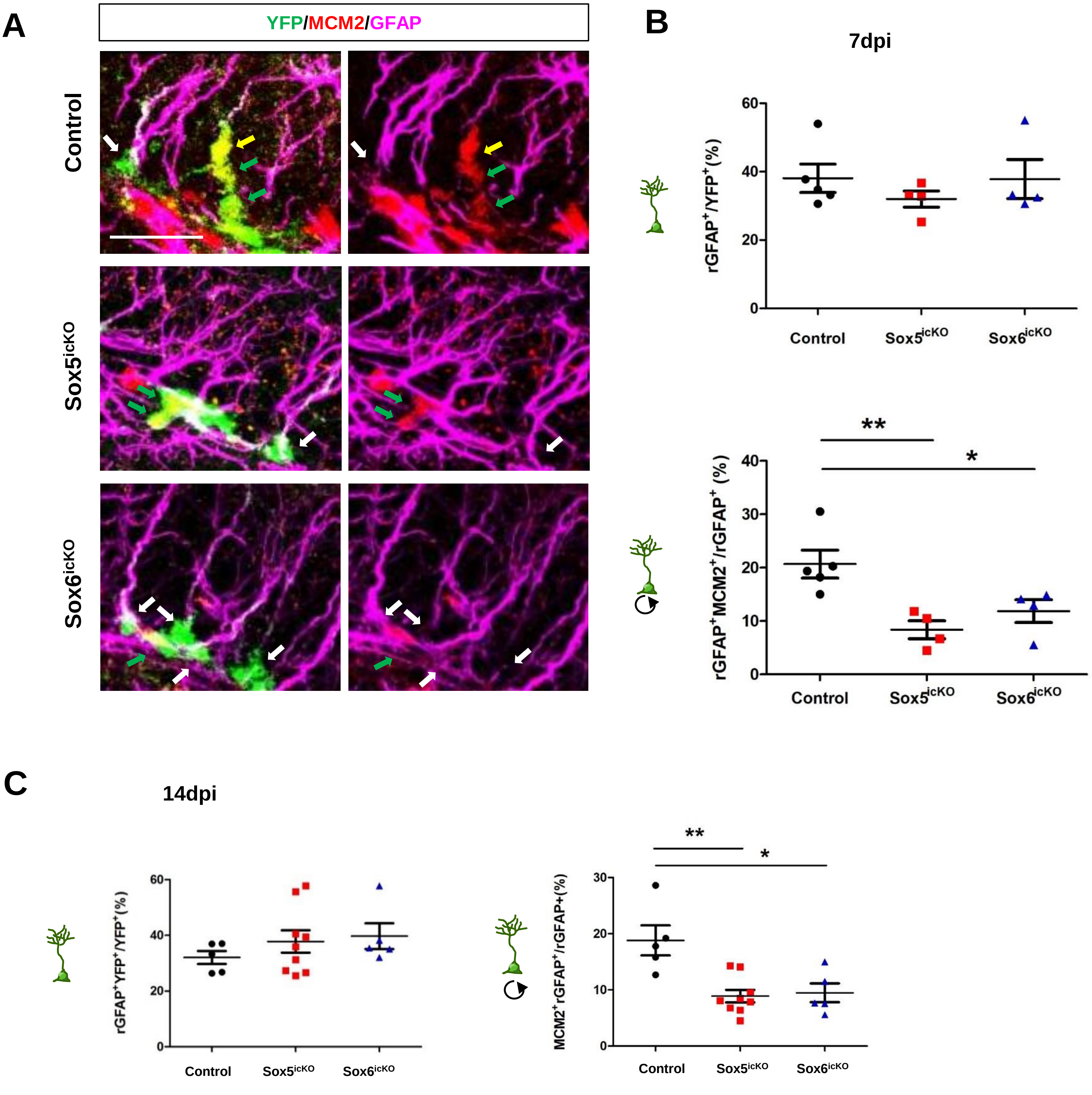

Figure S3. Sox5 and Sox6 are required for the activation of adult RGLs at 7 and 14dpi. Related to Figure 2. (**A**) Confocal images showing YFP, MCM2 and radial (r) GFAP at 7dpi in adult Control, $Sox5^{icKO}$ and $Sox6^{icKO}$ mice. White arrows indicate $YFP^{+}rGFAP^{+}MCM2^{-}$ cells, green arrows $YFP^{+}MCM2^{+}$ cells and yellow arrows $YFP^{+}rGFAP^{+}MCM2^{+}$ cells. **(B,C)** Quantitation of the number of $rGFAP^{+}$ cells in YFP^{+} cells and number of double $rGFAP^{+}$ $MCM2^{+}$ cells in recombined $rGFAP^{+}$ YFP^{+} cells in Control, $Sox5^{icKO}$ and $Sox6^{icKO}$ mice at 7dpi (B) and 14dpi (C). Data represents mean values ± SEM. Unpaired two-tailed Student's t test comparing each group with Control, *$p<0.05$, **$p<0.01$, *** $p< 0.001$. Scale bar, 25 µm.

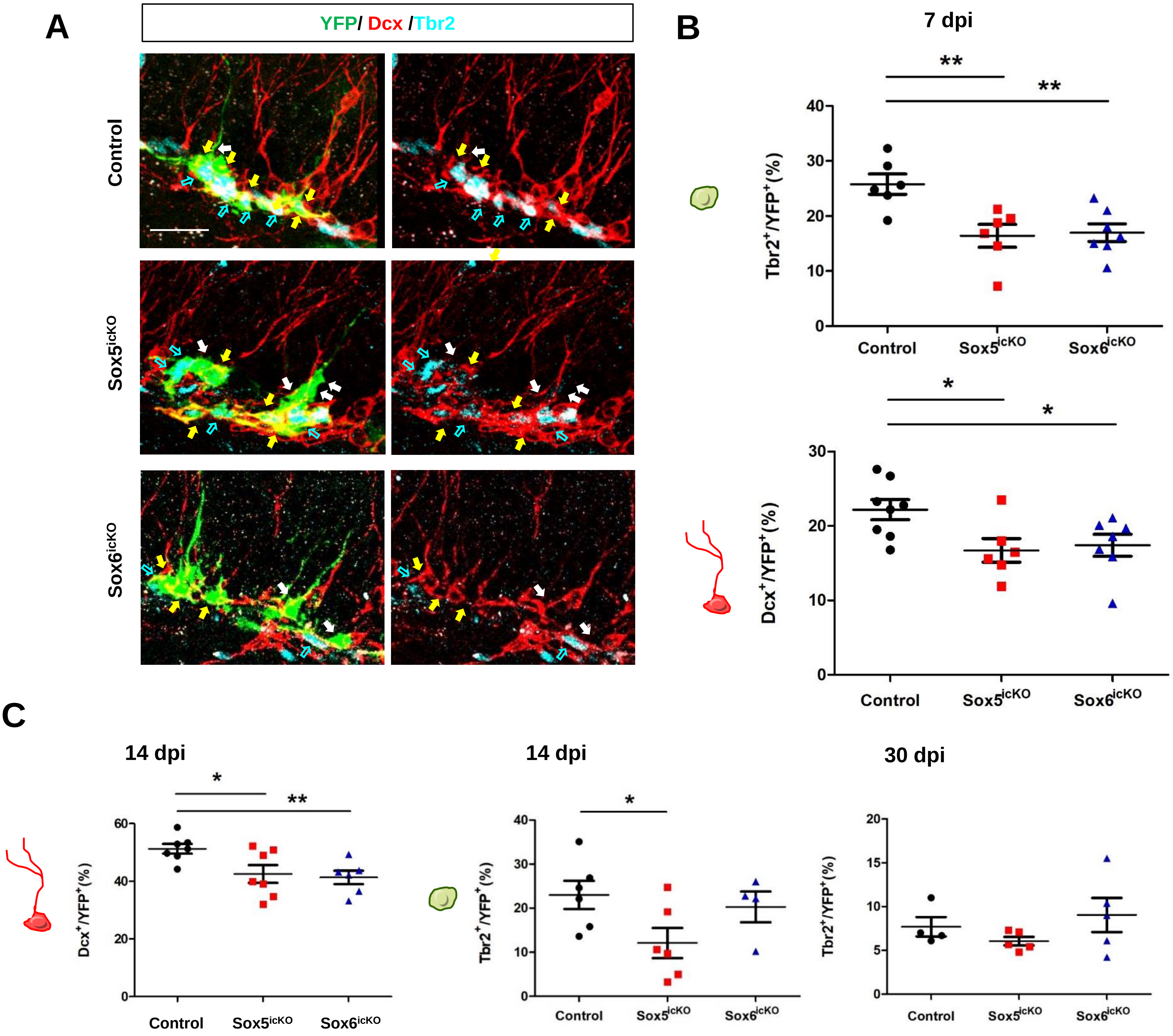

Figure S4. Sox5 and Sox6 are required for the generation of newborn neurons 7dpi and 14dpi and are not essential for astrogliogenesis. Related to Figure 4. (A) Confocal images showing YFP, Dcx and Tbr2 in the SGZ at 7dpi in 2-month old Control, $Sox5^{icKO}$ and $Sox6^{icKO}$ mice. Cyan arrows indicate $Tbr2^{+}$ YFP^{+} cells, yellow arrows Dcx^{+} YFP^{+} cells and white arrows $YFP^{+}Tbr2^{-}Dcx^{-}$ cells. **(B, C)** Quantitation of $Tbr2^{+}$ and Dcx^{+} cell number in the population of YFP^{+} cells in the indicated mice after 7 (B), 14dpi (C) or 30dpi (C, right). Data represents mean values ± SEM. Unpaired two-tailed Student's t test comparing each group with Control, *p<0.05, **p<0.01 , *** p< 0.001. Scale bar represents 25 µm.

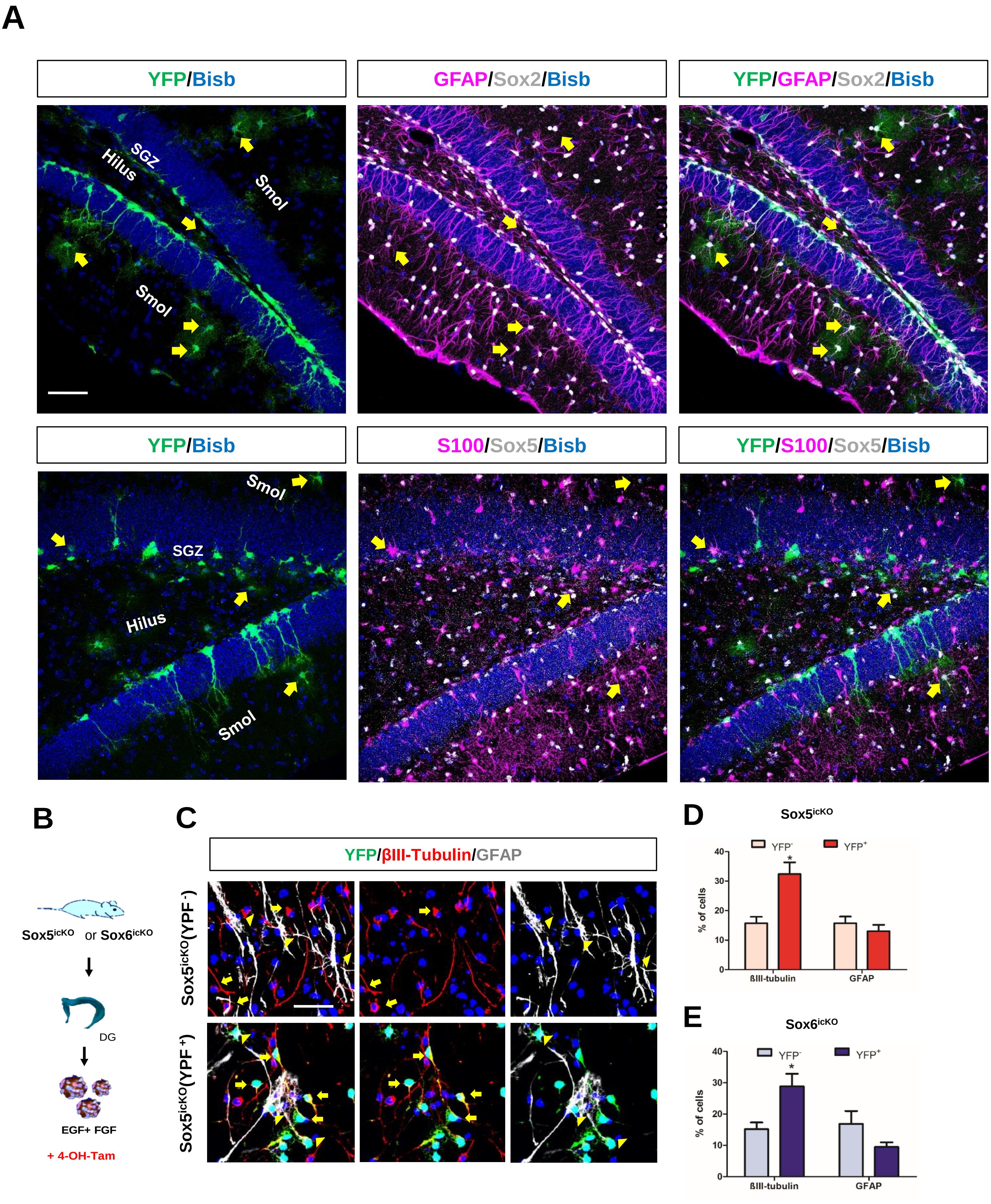
A
YFP/Bisb
GFAP/Sox2/Bisb
YFP/GFAP/Sox2/Bisb
SGZ
Hilus
Smol
Smol
YFP/Bisb
S100/Sox5/Bisb
YFP/S100/Sox5/Bisb
Smol
SGZ
Hilus
Smol
B
Sox5icKO or Sox6icKO
DG
EGF+ FGF
+ 4-OH-Tam
C
YFP/βIII-Tubulin/GFAP
Sox5icKO(YPF-)
Sox5icKO(YPF+)
D
Sox5icKO
YFP-
YFP+
% of cells
ßIII-tubulin
GFAP
E
Sox6icKO
YFP-
YFP+
% of cells
ßIII-tubulin
GFAP

Figure S5. Sox2-creERT2 line drives cre mediated recombination in astrocytes. Loss of Sox5 or Sox6 promotes neuronal fate in adult NSCs *in vitro*. Related to Figure 4. (A) Confocal images of the SGZ showing inmunostaining in Control Sox2-creERT2/Rosa26-YFP mice 7 days upon TAM-induced recombination. Yellow arrows indicate $YFP^{+}/Sox2^{+}/GFAP^{+}$ or $YFP^{+}/Sox5^{+}/S100\beta^{+}$ cells in the Hilus (Hi) and Stratum moleculare (Smol). Subgranular zone (SGZ). **(B)** Neurospheres obtained from 8-weeks old hippocampus of $Sox5^{icKO}$ and $Sox6^{icKO}$ mice grown in adherent conditions and induced to perform cre-recombination adding 4-OH-TAM (100nM) for 4 days. **(C)** Inmunostaining for YFP, βIII-tubulin and GFAP in not recombined (YFP^{-}) and in recombined (YFP^{+}) cells. Differentiated neurons (yellow arrows) and astrocytes (yellow arrowheads) are indicated. Nuclei were stained with bisbenzimide (blue). **(D,E)** Quantitation of the percentage of βIII-tubulin^{+} differentiated neurons and $GFAP^{+}$ astrocytes in the population of YFP^{+} or YFP^{-} cells (n > 100 each) obtained from $Sox5^{icKO}$ (E) or $Sox6^{icKO}$ (F) mice. Data represent mean values ± SEM of three independent experiments. * P <0.05=; ** P <0.005 *** P < 0.001 according to a paired Student's t-test. Bar is 50 µm in A and 15 µm in C.

A

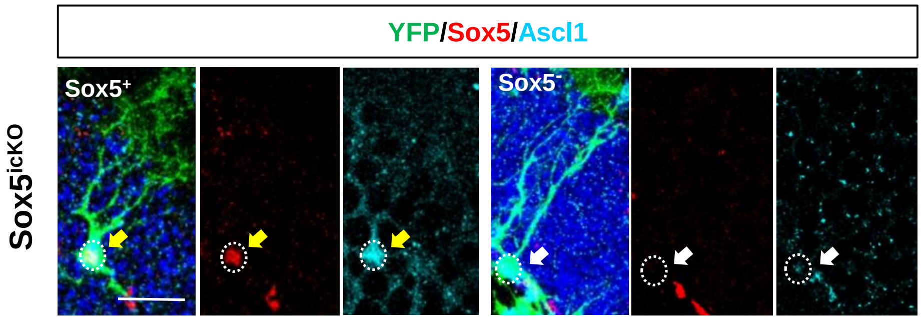

Figure S6. Ascl1 expression is lost from RGLs where Sox5 is conditionally deleted. Related to Figure 5. (A) Confocal images showing RGLs in the SGZ of 3 month-old Sox5^{icKO} mice at 14dpi using immunohistochemistry for YFP, Sox5 and Ascl1. Yellow arrows show a YFP^{+}Sox5^{+}Ascl1^{+} RGL and white arrows a YFP^{+}Sox5^{-}Ascl1^{-} RGL. Bar represents 20 µm.
